# Chromatin fiber breaks into clutches under tension and crowding

**DOI:** 10.1093/nar/gkac725

**Published:** 2022-08-27

**Authors:** Shuming Liu, Xingcheng Lin, Bin Zhang

**Affiliations:** Department of Chemistry, Massachusetts Institute of Technology, Cambridge, MA, USA; Department of Chemistry, Massachusetts Institute of Technology, Cambridge, MA, USA; Department of Chemistry, Massachusetts Institute of Technology, Cambridge, MA, USA

## Abstract

The arrangement of nucleosomes inside chromatin is of extensive interest. While in vitro experiments have revealed the formation of 30 nm fibers, most *in vivo* studies have failed to confirm their presence in cell nuclei. To reconcile the diverging experimental findings, we characterized chromatin organization using a residue-level coarse-grained model. The computed force–extension curve matches well with measurements from single-molecule experiments. Notably, we found that a dodeca-nucleosome in the two-helix zigzag conformation breaks into structures with nucleosome clutches and a mix of trimers and tetramers under tension. Such unfolded configurations can also be stabilized through trans interactions with other chromatin chains. Our study suggests that unfolding from chromatin fibers could contribute to the irregularity of *in vivo* chromatin configurations. We further revealed that chromatin segments with fibril or clutch structures engaged in distinct binding modes and discussed the implications of these inter-chain interactions for a potential sol–gel phase transition.

## INTRODUCTION

Eukaryotic genomes are packaged into nucleosomes by wrapping DNA around histone proteins. While the structure of a single nucleosome has been extensively characterized (1–3), the organization for a string of nucleosomes, that is, chromatin, remains debatable (4–6). Regular, fibril configurations are commonly observed in experiments that study chromatin materials extracted from the nucleus (7–10). The invention of in vitro reconstituted nucleosome arrays with strong-positioning DNA sequences (11) helped to remove sample heterogeneity in nucleosome spacing and made possible the determination of high-resolution structures (12–16). However, despite the large amount of evidence supporting their formation in vitro, fibril structures are rarely detected by in vivo experiments that have managed to characterize chromatin at a fine resolution (17–20). Therefore, their biological relevance has been questioned, and chromatin organization inside the nucleus remains controversial.

It is worth noting that the nuclear environment is rather complex. In addition to interactions among nucleosomes, many factors, including tension and crowding, can impact chromatin organization. Chromatin is known to associate with various force-generating protein molecules involved in transcription and nucleosome remodeling (21–25). Furthermore, chromatin is often attached to the nuclear envelope and other liquid droplet-like nuclear bodies (26–29). Dynamical fluctuations in these nuclear landmarks could exert forces on chromatin as well (30,31). Finally, local nucleosome density can be quite high, especially in heterochromatin regions (32). Such a crowded environment could lead to cross-chain contacts that might compete with interactions stabilizing single-chain conformations (17). Therefore, both tension and crowding could destabilize the most stable configuration for isolated chromatin, driving chromatin unfolding and the formation of irregular structures.

Chromatin unfolding has indeed been studied extensively with various techniques (33,34). Single-molecule force spectroscopy is a powerful tool for characterizing chromatin organization under tension (35–38). Force–extension curves at low-force regimes are particularly informative regarding inter-nucleosomal interactions (39). Single-molecule Förster resonance energy transfer is another popular technique for probing nucleosome contacts and chromatin conformational dynamics (40–43). Mesoscopic modeling has also been frequently used to interpret experimental data with structural details (44–51). However, because of the experimental techniques’ low resolution and assumptions on nucleosome-nucleosome interactions introduced in computational models, the exact conformations of unfolded chromatin have not reached a consensus and necessitates further investigations.

We perform computer simulations of a 12-nucleosome-long chromatin segment (12mer) to investigate chromatin unfolding under tension and crowding. Residue-level coarse-grained representations are adopted for protein and DNA molecules to capture their interactions with physical chemistry potentials at high resolution. Using a combination of enhanced sampling techniques and machine learning, we show that the computed force–extension curve agrees well with results from single-molecule force spectroscopy experiments (52). Our simulations support chromatin unfolding under tension proceeds through intermediate structures with nucleosome clutches, that is, configurations that have been directly observed via super-resolution imaging of cell nucleus (53). These structures sacrifice nucleosomal DNA by unwrapping to preserve close contacts among neighboring nucleosomes. In addition, the presence of another 12mer promotes inter-chain interactions to stabilize extended chromatin configurations as well. Together, our results suggest that in vivo chromatin configurations can arise from the unfolding of fibril configurations as a result of tension and crowding.

## MATERIALS AND METHODS

### Coarse-grained modeling of chromatin organization

We applied a coarse-grained model to study a chromatin segment with twelve nucleosomes. The structure-based model (54,55) was used to represent protein molecules with one bead per amino acid and stabilize the tertiary structure of the histone octamer while maintaining the conformational flexibility of disordered tail regions. Secondary structure motifs in the disordered regions of histone proteins do not impact nucleosome stability and protein-DNA interactions (Supplementary Figure S1) and were not explicitly accounted for in the model. Protein molecules from different nucleosomes interact through both an electrostatic and amino acid-specific potential (56). We represented the DNA molecule with three beads per nucleotide using the 3SPN.2C model (57). Protein-DNA interactions were described with the screened Debye-Hückel potential at a salt concentration of 150 mM and the Lennard-Jones potential for excluded volume effect. We ignored electrostatics interactions for particles that are farther than four times the screening length (3.14 nm), at which point the Debye–Hückel potential is expected to become negligible. Further increasing the cutoff length does not quantitatively impact simulation results (Supplementary Figure S2). The coarse-grained model has been used extensively in prior studies with great success to investigate protein-protein/protein-DNA interactions (58,59), the energetics of single nucleosome unwinding (60,61), nucleosome-nucleosome interactions (62), and the folding pathways of a tetra-nucleosome (63). More details on the model setup and validation and force field parameters can be found in the Supporting Information.

The software package LAMMPS (64) was used to perform molecular dynamics simulations with periodic boundary conditions and a time step of 10 fs. The length of the cubic simulation box was set as 2000 nm, which is much larger than the maximum chromatin extension length to prevent interactions between periodic images. We used the Nosé-Hoover style algorithm (65) to maintain the temperature at 300 K with a damping constant of 1 ps. The globular domains of histone proteins and the inner layer of nucleosomal DNA were rigidified. Positions and velocities of all the atoms within each rigid body were updated together such that the body moves and rotates as a single entity. Disordered histone tails, outer nucleosomal DNA, and linker DNA remained flexible, and no restrictions were applied to their conformational dynamics. Our partition of the rigid and flexible parts was motivated by prior studies showing that unwinding the inner layer of nucleosomal DNA does not occur at forces below 5 pN (39,52). Furthermore, the histone core remains relatively stable during DNA unwinding (66,67). As shown in Supplementary Figure S11 of (63), this treatment does not impact the accuracy in sampling inter-nucleosome interactions but significantly reduces the computational cost. Rigidizing inner layer DNA with the globular domains of histone proteins does not affect the energetics of outer layer DNA unwrapping either (Supplementary Figure S3).

### Force extension curves from enhanced sampling

To characterize chromatin structures under tension and compute force–extension curves, we introduced two collective variables that monitor the important degrees of freedom for chromatin unfolding. The first variable, *d*_stack_, measures the average geometric center distance between the *i*th and (*i* + 2)th nucleosomes. For small values of *d*_stack_, nucleosomes are stacked on top of each other as in the zigzag conformation (13,68). The second variable, *q*_wrap_, quantifies the average degree of nucleosome unwrapping. The two variables can better differentiate the various chromatin conformations and capture the energetic cost of extension than the DNA end-to-end distance. Mathematical expressions for the two variables are provided in the Supporting Information.

For extension forces below 3 pN, we carried out a set of two-dimensional umbrella sampling based on *q*_wrap_ and *d*_stack_. *q*_wrap_ was restricted to centers from 0.45 to 0.90 with a spacing of 0.15 and a spring constant of 50.0 kcal/mol. *d*_stack_ was limited to centers from 10.0 to 30.0 nm with a spacing of 5 nm and a spring constant of 0.05 kcal/(mol · nm^2^). Additional simulations were added to improve the overlap between umbrella windows and the convergence of free energy calculations. When extension forces are larger than 3 pN, chromatin can adopt fully unstacked structures with large end-to-end distances. Covering the entire accessible phase space with two-dimensional umbrella simulations becomes too costly computationally. Therefore, we restricted to one-dimensional free energy calculations using *d*_stack_ as the collective variable.

Most simulations were initialized from the most probable configurations predicted by a neural network model for chromatin stability under the same umbrella biases (see below and Supporting Information for details). They lasted for at least 10 million steps. Details of the umbrella centers and spring constants used in simulations and exact trajectory lengths are provided in Supplementary Table S1. We computed the error bars by dividing the data into three equal-length, non-overlapping blocks and calculated the respective quantities using data from each block. The standard deviations of the three estimations were used to measure the errors of the mean.

### Facilitating conformational sampling with a neural network model for chromatin

Conformational sampling for the 12mer is challenging because of the many possible degenerate configurations. For example, both unstacking and unwrapping can extend chromatin, and different combinations of the two from various nucleosomes can result in many structures that share similar end-to-end distances. Conformational transitions are slow due to considerable energetic barriers arising from non-specific electrostatic interactions.

To alleviate the sampling problem, we introduced a neural network model for the 12mer. As detailed in the Supporting Information, the model quantifies the stability and the free energy of chromatin structures using inter-nucleosome distances (Supplementary Figure S4). It was parameterized using mean forces estimated with coarse-grained simulations for 10 000 independent tetra-nucleosome configurations (63). The neural network model is computationally efficient and allows exhaustive Monte Carlo sampling to determine the most likely chromatin structures at a given setup. These structures were provided to initialize coarse-grained simulations and free energy calculations.

The neural network model is imperfect due to approximations introduced when building the free energy surface with tetra-nucleosome calculations. However, it does reproduce the force–extension curve reasonably well at the lower force regime (Supplementary Figure S5). We only used the neural network model for conformational exploration, and all quantitative results presented in the manuscript were obtained with coarse-grained simulations.

### Exploring the impact of crowding on chromatin extension

To study the effect of crowding on chromatin organization, we computed the free energy profile as a function of two collective variables that measure intra- and inter-chain contacts. Umbrella sampling was used to enhance conformational exploration, and details on the restraining centers and constants are provided in Supplementary Table S2.

Umbrella simulations were initialized from configurations in which the two chains were separated far apart with zero contacts. For simulations biased toward small values of $\bar{d}_\mathrm{stack}< 10$ nm, we prepared each chromatin with a two-helix zigzag configuration that resembles the cryo-EM structure (13). The rest of the simulations were initialized with extended chromatin configurations predicted by the neural network model. More simulation details can be found in the Supporting Information.

## RESULTS

### Coarse-grained modeling reproduces force–extension curve

**Figure 1.**
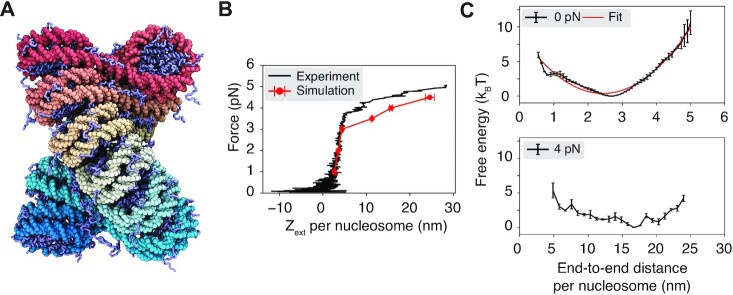
Coarse-grained modeling reproduces the force–extension curve for chromatin. (**A**) Illustration of the two-helix fibril chromatin structure with a linker length of 20 bp. The DNA molecule varies from red to cyan across the two ends, and histone proteins are drawn in ice blue. (**B**) Comparison between the simulated (red) and experimental (52) (black) force–extension curve. (**C**) Free energy profiles as a function of the DNA end-to-end distance per nucleosome computed with the presence of 0 pN (top) and 4 pN (bottom) extension force. A harmonic fit to the 0 pN simulation result is shown in red. Error bars correspond to the standard deviation of the mean estimated via block averaging by dividing simulation trajectories into three independent blocks of equal length.

We applied a residue-level coarse-grained model to characterize the unfolding of a 12mer chromatin with the 601 nucleosome positioning sequence (11) and a linker length of 20 bp (Figure 1 A). One bead per amino acid and three sites per nucleotide were employed to describe protein and DNA molecules, leading to a system of 23 590 coarse-grained beads in size. Interactions among the coarse-grained beads were parameterized by accounting for solvent effect implicitly with physically motivated potentials (see Materials and Methods for model details). Similar approaches have been extensively used to characterize single nucleosomes (60,62,69) and nucleosome oligomers (63,70) with great success.

We computed the average chromatin extension length under various pulling forces along the $z$-axis for a direct comparison with results from single-molecule pulling experiments (52). Comprehensive sampling of chromatin conformations can be rather challenging because of the non-specific and strong electrostatic interactions between nucleosomes that give rise to slow dynamics. To alleviate the sampling difficulty, we carried out umbrella simulations (71) on two collective variables that quantify the degree of nucleosomal DNA unwrapping (*q*_wrap_) and nucleosome unstacking (*d*_stack_) (Supplementary Figure S6). The simulations were initialized from the most probable configurations at respective umbrella centers obtained from an exhaustive sampling of a neural network model that approximates the free energy landscape of the 12mer in terms of inter-nucleosome distances (see Materials and Methods). This initialization protocol attempts to prepare umbrella simulations with equilibrium configurations to avoid traps of local minima.

As shown in Figure 1B, the simulation results match well with the experimental force–extension curve measured by Kaczmarczyk *et al.* (52). In particular, we observe a linear extension regime at low forces (≤3 pN). The sharp increase in extension at large forces deviates from the linear behavior, resulting in a plateau regime. We emphasize that there are no tuning parameters in the model, and we do not make assumptions regarding stacking energies.

The free energy profiles as a function of the DNA end-to-end distance are consistent with the linear and plateau regimes seen in force–extension curves (Figure 1 C). In particular, at 0 pN force, the free energy curve can be well approximated with a harmonic potential, which naturally produces a linear relationship between the force and extension. Consistent with a harmonic behavior near the minimum, theoretical predictions based on the free energy profile at 0 pN match well with simulation results at 1–3 pN (Supplementary Figure S7). However, the free energy profile at 4 pN is strongly anharmonic. The bottom panel shows that the curve is relatively flat over a wide range of end-to-end distances. Because of the lack of energetic penalty, a slight change in pulling force can produce significant variations in the extension length, giving rise to the observed plateau regime.

We note that several factors could contribute to the discrepancy between simulated and experimental curves at large forces. For example, the relatively flat landscape over a wide range of chromatin conformations makes it challenging to predict the free energy minimum and the average end-to-end distance. Minor errors in conformational sampling and free energy calculations could be amplified into significant changes in the chromatin extension. In addition, our implicit treatment of counter ions introduces approximations to protein–DNA interactions. It may be insufficient to mimic the exact experimental setting with both monovalent and divalent ions (52). Consistent with this hypothesis, varying the salt concentration in simulations improved the agreement in the average extension length with the experimental value (Supplementary Figure S8).

### Intermediate states support nucleosome-clutch formation

The nucleosome arrangement in extended, unfolded chromatin has been the subject of numerous studies (36,38,43,46). The residue-level coarse-grained simulations offer a unique opportunity to produce high-resolution structures with minimal assumptions. Their success in reproducing experimental observations shown in Figure 1 B and Supplementary Figure S8 supports the biological relevance of the predicted structures.

**Figure 2.**
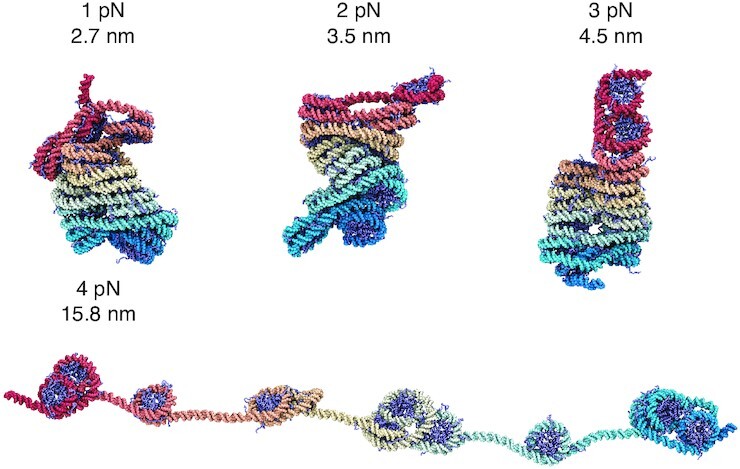
Representative chromatin structures from simulations performed under various extension forces (also see Supplementary Figure S9). The values for the extension force and the end-to-end distance per nucleosome are provided next to the structures. The same coloring scheme as in Figure 1 A is adopted here.

We determined representative structures at various forces to better characterize chromatin unfolding under tension (Figure 2). These structures share end-to-end distances close to the mean force-dependent extension lengths. They correspond to the central configurations of the most populated clusters identified by the single-linkage algorithm (72) using root mean squared distance (RMSD) as the distance between structures. At small forces (≤3 pN), though chromatin extends linearly, we do not observe a uniform extension of nucleosomes along the principal fiber axis (Figure 2 and Supplementary Figure S9). The conformational change mainly occurred in the plane perpendicular to the fiber axis via a shearing motion, causing the formation of irregular, compact structures. Such structures are more kinetically accessible as they avoid complete unstacking, which could cause a significant energetic penalty as shown by Moller *et al.* (62). We note that an ensemble of chromatin configurations exists at a given end-to-end distance, and only example ones are shown in Figure 2. Averaging the entire ensemble produces more symmetric structures and nucleosome contact patterns (Supplementary Figure S10).

**Figure 3.**
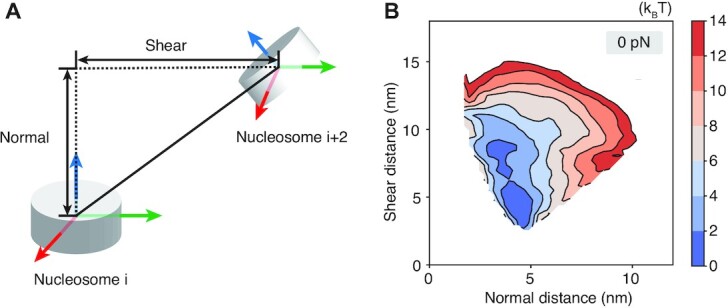
Chromatin extension favors shearing motion within the nucleosomal plane over the normal motion perpendicular to the plane. (**A**) Illustration of the nucleosome coordinate system and the decomposition of the inter-nucleosome distance into shearing and normal components. (**B**) The free energy profile as a function of the two different modes of breaking inter-nucleosome distances shown in part A.

The preference of shearing over complete unstacking can be readily seen in Figure 3. There, we decomposed the distance between two nucleosomes into motions that are within or perpendicular to the nucleosomal plane (Supplementary Figure S11). We further computed the free energy profile for the two decomposed distances under no extension force. It is evident that the energetic penalty for chromatin unfolding along the shearing direction is much smaller. Shearing can better preserve inter-nucleosome contacts as nucleosomes move away from each other, lowering the energetic penalty.

**Figure 4.**
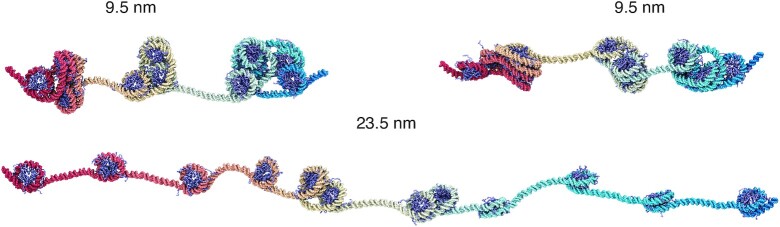
Representative chromatin structures at smaller and larger distances than the average extension at 4 pN force (see also Supplementary Figure S12). These structures again support the formation of nucleosome clutches, which do not break into individual nucleosomes until at very large per-nucleosome end-to-end distances around 23.5 nm.

The representative structure from 3 to 4 pN undergoes a dramatic transformation from a compact configuration to one with many nucleosomes losing stacking interactions. Notably, the unfolded structures fall into small clusters of nucleosomes. These structures often feature one or two nucleosomes with a highly unwrapped outer layer. Unwrapping the outer layer DNA only incurs modest energetic cost (60,61,73) and serves as an economic strategy to extend chromatin under force. Nucleosome clutch formation is not specific to a particular end-to-end distance and can be readily seen in structures with smaller distances as well (Figure 4 and Supplementary Figure S12). We note that the nucleosomes that remain in contact are not perfectly stacked as in the crystal structure of a tetranucleosome (68), but are somewhat irregular as configurations observed in prior simulations (49,63) and *in vivo* experiments (19,74–76). Further stretching the chromatin eventually leads to configurations with most of the outer nucleosomal DNA unwrapped.

Our results suggest chromatin unfolding does not proceed via a uniform nucleosome unstacking. On the contrary, nucleosomes prefer to stay in close contact as much as possible by forming clusters separated by unwrapped DNA. To ensure that the formation of nucleosome clutches is not a result of biases from initial configurations prepared by the neural network model, we carried out an additional set of simulations starting from uniformly extended chromatin structures (Supplementary Figure S13A). More simulation details are provided in the Supporting Information and Supplementary Tables S3 and S4. As shown in Supplementary Figure S14A, these new simulations produced a free energy profile as a function of the end-to-end distance that matches well with the one presented in Figure 1C, supporting the statistical convergence of our simulations. To resolve the degree of clutch formation in chromatin configurations, we introduced a new collective variable, α, that quantifies the ratio of the maximum and minimum distance between 1 and 3 nucleosomes, that is, $\alpha = d_{i,i+2}^\text{max}/d_{i,i+2}^\text{min}$. For clutched configurations, the distance between two nucleosome clusters is expected to be much larger than the distance between nucleosomes within the same cluster, and α will be much larger than one. On the other hand, for more uniformly extended configurations, α will approach one. The free energy profile as a function of α exhibits a global minimum at values much larger than one (Supplementary Figure S14B), supporting the stability of clutched configurations. Example configurations at various end-to-end distances adopt large α values (Supplementary Figure S13B) and resemble those presented in Figure 2. Therefore, the formation of nucleosome clutches under tension is an inherent property of chromatin and robust to simulation protocols.

We further confirmed that the unwrapping of the outer nucleosomal DNA is essential for clutch formation. In a new set of umbrella simulations, we removed DNA unwrapping by rigidifying the entire 147 bp nucleosomal DNA with the histone core. These simulations were performed with the presence of 4 pN force and were initialized from the fibril structure (see the Supporting Information for additional simulation details). Supplementary Figure S15 shows that, when the DNA was prohibited from unwrapping, chromatin favors more uniform configurations when extended. The free energy profile as a function of α computed with the new simulations reaches the minimum value at around 3 (Supplementary Figure S15B). On the other hand, much larger values for α are favored when unwrapping is allowed. DNA unwrapping helps chromatin preserve inter-nucleosome contacts when stretched, leading to energetically more favorable clutched configurations (Supplementary Figure S16). Without unwrapping, inter-nucleosome contacts must be broken to satisfy geometric constraints to reach a given extension, resulting in more uniform chromatin structures (Supplementary Figure S15C).

### Inter-chain contacts stabilize unfolded chromatin

The pulling simulations suggest that *in vivo* configurations can arise from the unfolding of chromatin fiber under tension. Inside the nucleus, chromatin is not in isolation but surrounded by other chromatin segments in a crowded environment (20,53). The more exposed nucleosomes in the intermediate configurations could facilitate inter-chain interactions, further stabilizing the unfolded structures.

**Figure 5.**
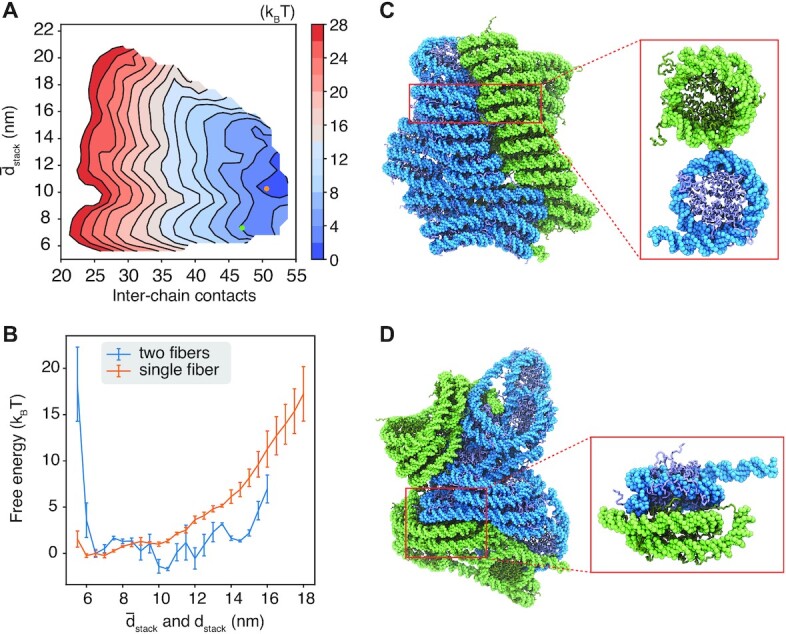
Crowding and inter-chain contacts stabilize extended chromatin configurations. (**A**) The free energy surface as a function of the inter-chain contacts and the average extension of the two 12mers. (**B**) Free energy profiles of chromatin unstacking with (blue) and without (orange) the presence of an additional 12mer. Chromatin unstacking is quantified with *d*_stack_ and $\bar{d}_{\text{stack}}$ for single and two fiber simulations, respectively. (**C**) Representative structure for two contacting chromatin segments that maintain fibril configurations, with the corresponding collective variables indicated as the green dot in part A. The inset highlights the side-side contacts between inter-chain nucleosomes. (**D**) Representative structure of the free energy minimum, with the corresponding collective variables indicated as the orange dot in part A. The inset highlights the stacking interactions between inter-chain nucleosomes.

To evaluate the impact of crowding on chromatin stability, we computed a 2D free energy profile using simulations with two 12mers. The first collective variable quantifies the inter-chain contacts as the number of nucleosome pairs within a distance of 15 nm. Only pairs with one nucleosome from each chromatin segment were included to define the contacts. The other dimension measures chromatin extension using the average unstacking of the two chains, $\bar{d}_\text{stack}$. Figure 5 A shows that configurations with close contacts between the two chromatin segments are more favorable. A representative structure for two contacting fibril chromatin identified by the single-linkage clustering algorithm is provided in Figure 5C. The contacts are mediated mainly by histone tail–DNA interactions, as can be seen in the inset that provides a zoomed-in view of the interface. Favorable interactions for compact chromatin are consistent with previous simulation studies that support the liquid chromatin state (77).

Notably, the global minimum of the free energy profile resides at larger values for $\bar{d}_\text{stack}$ corresponding to more extended chromatin configurations. While extending chromatin is unfavorable (Figure 5B), such structures promote close contacts between nucleosomes from different chains (Figure 5D). In particular, trans-nucleosomes can now engage in stacking interactions (Figure 5D), which are more favorable energetically compared to side-side contacts (62). The emergence of a new binding mode, unavailable when chromatin is constrained into fibril configurations, compensates for the energetic penalty of breaking cis-chain contacts. Further extending the chromatin leads to more intertwined structures at a rather modest energetic cost (Supplementary Figure S17).

Similar to the single-chain simulations, extending chromatin again led to irregular configurations with nucleosome clutches. As shown in Supplementary Figures S18 and S19, the degree of irregularity increases monotonically with $\bar{d}_\text{stack}$ and for intermediate values of inter-chain contacts. DNA unwrapping in irregular chromatin configurations relieves the torsional constraints on nucleosomes to sample a much wider range of relative nucleosome-nucleosome orientations and distances. As a result, nucleosomes can now engage in many simultaneous energetically-favorable interactions, both with nucleosomes from the same chain and different chains.

To further evaluate the contribution of DNA unwrapping to inter-chain contacts, we carried out a new set of simulations with fully rigidified nucleosomes. As before, the core nucleosomes move as rigid bodies, and only linker DNA and histone tails were kept flexible. The setup of umbrella centers and restraining constants are similar to simulations that allow DNA unwrapping (Supplementary Table S5), and more details are provided in the Supporting Information. We found that the two chromatin forms fewer contacts in the new simulations. The free energy minimum for inter-chain contacts is located around 42 (Supplementary Figure S20), a value that is much smaller than that shown in Figure 5. Chromatin is less extended when DNA unwrapping is prohibited, reducing the free energy minimum for $\bar{d}_{\text{stack}}$ from 10 to 8 nm. When chromatin does extend, the configurations are also more uniform with less irregularity (Supplementary Figure S20), hindering the formation of interdigitated structures.

## CONCLUSIONS AND DISCUSSION

We characterized the impact of tension and crowding on chromatin organization with computational modeling using a coarse-grained model. The compact fibril configuration with nucleosomes following a zigzag path was most stable for a 12mer chromatin segment in isolation. Consistent with previous studies (45,46,50,78), we observed both unwrapping of nucleosomal DNA and unstacking between nucleosomes as chromatin unfolds from the fibril configuration due to the presence of tension. However, these changes are non-uniform and are initially localized to a small set of nucleosomes, leading to the formation of nucleosome clutches separated by unwrapped nucleosomal DNA. Such intermediate structures emerge as a result of balancing intra- and inter-nucleosome interactions. The clutched configurations sacrifice nucleosomal DNA by unwrapping to extend chromatin and preserve the energetically more favorable inter-nucleosome contacts.

Notably, the simulated intermediate structures resemble in vivo chromatin configurations. For example, super-resolution imaging of the core histone protein H2B in interphase human fibroblast nuclei has revealed the formation of nucleosome clutches of varying size (53). High-resolution electron tomography studies further support the prevalence of trimers in the clutches (19,75). Cross-linking-based experiments that detect nucleosome contacts in situ support nucleosome clutches with tri- or tetranucleosome as well (74,79). Our results generalize the findings from a previous study on tetra-nucleosomes (63). They support that certain in vivo chromatin structures may form as a result of unfolding from the fibril configuration. Since chromatin inside the nucleus can experience forces from various active processes (21,22,80–83), even for DNA sequences and linker lengths that strongly favor the fibril structure, chromatin may adopt irregular configurations because of tension. The clutched configurations are mostly seen at forces below 4 pN, a value that is indeed within the range expected from molecular motors (21,81–83).

We further showed that unfolded chromatin could promote inter-chain contacts, leading to the formation of interdigitated structures. Such structures present an alternative binding mode compared to the close contacts between two fibril configurations. Interdigitation is indeed consistent with electron microscopy images of two chromatin segments that are in close contact (84–90). These images revealed structures with diameters less than twice the 30 nm fiber, supporting an overlap between the two chromatin. In addition to supporting chromatin unfolding in a crowded environment, the interdigitated structures suggest that chromatin may, in fact, form gels at high density inside the nucleus. Gelation can form due to the stacking interactions between exposed nucleosomes from different chains, which are stronger than side-side interactions that are only accessible for nucleosomes in closely stacked fibers. The emergence of strong interactions could arrest the coarsening dynamics of small clusters to drive the percolation transition (91). Furthermore, interdigitation could give rise to topological entanglements among chromatin chains, further producing slow kinetics and gelation. Therefore, the two binding modes could help understand the observation of both liquid and gel state of chromatin mixtures (76,77,92–95).

We studied idealized chromatin with uniform DNA linker length and strong positioning sequence. Nucleosomes from natural chromatin are more heterogeneous with variations in histone modifications (96–100), linker DNA lengths and DNA sequences (12,70,101–105), and linker histone binding (13,53,106–108). Such heterogeneity could also contribute to the formation of irregular chromatin structures and clutches, as shown recently by the Schlick group (100,104,109). Our findings complement these studies and point to additional intrinsic factors that affect the stability of chromatin fibers. They might be particularly relevant for interpreting chromatin organization in heterochromatic regions and mitotic chromosomes. Due to its low transcriptional activity, chromatin in these systems is expected to be more uniform in histone modifications and linker DNA length, and its irregular organization may indeed arise from tension and crowding effects.

## DATA AVAILABILITY

Data presented in this study is available upon reasonable request to the corresponding author.

## Supplementary Material

gkac725_Supplemental_File
