# Supplementary material for: Chromatin fiber breaks into clutches under tension and crowding
Source: Nucleic Acids Res. 2022 Aug 27;50(17):9738–47. doi: 10.1093/nar/gkac725 (PMC9508854; doi:10.1093/nar/gkac725)
Supplement: gkac725_Supplemental_File [file gkac725_supplemental_file.pdf]

Supporting Information: Chromatin fiber breaks into clutches under tension and crowding

Shuming Liu,[†,‡] Xingcheng Lin,[†,‡] and Bin Zhang[*,†]

†*Department of Chemistry, Massachusetts Institute of Technology, Cambridge, MA, USA*

‡*Contributed equally to this work*

E-mail: binz@mit.edu

Summary

Details of Coarse-grained Simulations **S-3**

System Setup S-3

Force Field Setup S-4

Free Energy Profiles for Chromatin Under Tension S-6

Initial configurations from the neural network model S-8

One-dimensional free energy calculations at 4.5 pN force S-9

Estimating the extension per nucleosome from experimental data S-10

Theoretical predictions of chromatin extension along the z-axis S-11

Decomposing Inter-nucleosome Distances into Shear and Normal Motions S-11

Free Energy Calculations for Two Interacting 12mers S-12

Neural Network Model for the 12mer Chromatin **S-13**

Parameterizing the Tetra-nucleosome Free Energy Landscape with Neural Networks S-14

Generalizing Tetra-nucleosome Results to 12mer Chromatin S-16
Numerical Simulations of the Neural Network Model S-17

Details of Validation Simulations **S-18**

Simulations Starting from Uniformly Extended Chromatin Configurations S-18
Simulations with Fully Rigidified Nucleosomes S-20
Chromatin extension under 4 pN force S-20
Inter-chain contacts with two 12mer simulations S-21

Details of Coarse-grained Simulations

We carried out all molecular dynamics simulations with the software LAMMPS.[S1] Umbrella sampling was performed using collective variables implemented by the Plumed package.[S2] We applied the weighted histogram method[S3,S4] and fastmbar[S5] to process the data and computed the unbiased extension length at a given force.

System Setup

We built a structural model for the chromatin with 12 nucleosomes and 20-bp linker DNA following two steps. We first connected 12 individual nucleosomes into a continuous segment without much regard to the overall chromatin topology. We then aligned the DNA model to a template that closely resembles the cryo-EM structure with a two-start fibril organization.[S6]

We connected individual nucleosomes to build a 12mer chromatin as follows. The nucleosome unit with 167-bp of DNA was extracted from the tetranucleosome X-ray structure (PDB ID:1ZBB).[S7] The DNA was taken as residues 158-324 of chain I and the corresponding complementary segment from chain J. There are no extra base pairs at the entry side of the nucleosome in this setup, but 20-bp linker DNA exists at the exiting end. The resulting sequence with the core DNA (147bp) in the underline is

ACAGGATGTAACCTGCAGATACTACCAAAAGTGTATTTGGAAACTGCTCCAT
CAAAAGGCATGTTCAGCTGGATTCCAGCTGAACATGCCTTTTGATGGAGCAG
TTTCCAAATACACTTTTGGTAGTATCTGCAGGTGATTCTCCAGGGCGGCCAG
TACTTACATGC

We further replaced the coordinates for histone proteins with that from PDB ID: 1KX5,[S8] which resolved the coordinates for histone tails.

We added one additional DNA base pair at the end of the linker DNA as one sticky end using the software 3DNA[S9] for alignment between neighboring nucleosomes. This 168-bp segment is the building block for constructing the dodecamer. For example, to extend

chromatin with n nucleosomes, we align the 168-th bp of the n-th nucleosome with the first bp of the $(n + 1)$-th nucleosome. The alignment determines the orientation of the $(n + 1)$-th nucleosome, and the fiber is extended by one nucleosome after removing the overlapping nucleotides. For the last (12-th) nucleosome, we deleted the linker DNA to build the dodecamer with 1984 bp of DNA. The resulting all-atom model was converted into the coarse-grained model with in-house scripts.

While the above procedure succeeds at building an all-atom model for the 12mer, the precise topology of the resulting structure cannot be controlled easily. To construct a two-start fibril configuration resembling the compact and twisted Cryo-EM structure,[S6] we aligned the model to a two-start fiber structure built by the software FiberModel, as detailed below. The structural alignment was performed using MDAnalysis[S10,S11] with RMSD coordinate fitting.[S12,S13]

The template was generated by the software fiberModel as the lowest energy configuration.[S14] FiberModel optimized fiber configurations by utilizing a series of geometric parameters, including the height per nucleosome along the fiber axis (h), the rotation angle per nucleosome around the fiber axis (θ), the radius of the fiber (R), and three Euler angles that determine the direction of each nucleosome (α, β, γ). To build a fibril chromatin structure, we set initial values for the parameters $(h, \theta, R, \alpha, \beta, \gamma)$ as (2.34 nm, 2.88, 7.3 nm, -3.14, 0.622, 0) as estimated from the cryo-EM structure.[S6] Also, each nucleosome was treated as a cylinder with a radius and height of 5.2 and 4.5 nm. We then used FiberModel to optimize the chromatin structure based on parameters α and γ, keeping the other parameters fixed. The optimization utilized the basin hopping global search technique.[S15] The final structure aligned with the FiberModel template is shown in Figure 1A.

Force Field Setup

We used the same force fields as in the tetra-nucleosome study[S16] to simulate the 12mer. The 3SPN.2C DNA model[S17] was adopted to model each nucleotide with three coarse-

grained beads for phosphate, sugar, and base, respectively. The Cα structure-based model[S18] was adopted to simulate the conformational dynamics of individual histone proteins. Both bonded and nonbonded interactions were generated based on the nucleosome crystal structure (PDB ID: 1KX5). For nonbonded contact potentials, two residues were considered in contact when their minimum distance is smaller than 6Å, implemented using the Shadow algorithm.[S19] We further scaled the default interaction strength[S4] by 2.5 to prevent proteins from unfolding at 300K. To model the disordered portions of the histones, we removed the dihedral and contact potentials for disordered residues not included in the core histones (residue ID: 44-135, 160-237, 258-352, 401-487, 531-622, 647-724, 745-839, 888-974). Including the secondary structure motifs in the disordered regions of histone proteins does not quantitatively change nucleosome stability and protein-DNA interactions (Figure S1). The IDs continuously index residues from chain A to chain H of the crystal structure with PDB ID: 1KX5.

In addition, residue-specific protein-protein interactions were introduced with the Miyazwa-Jernigan (MJ) potential[S20] and scaled by a factor of 0.4. In a previous study, we showed that the scaled MJ potential provides a balanced modeling of the radius of gyration for both folded and disordered proteins.[S16]

Protein-DNA interactions include the electrostatic potential modeled at the Debye-Hückel level with a monovalent salt concentration of 150 mM. In addition, a weak, non-specific Lennard-Jones potential was applied between all protein-DNA beads. Detailed expression for these potentials can be found in Ref. S21.

Our group and the de Pablo group have shown that the force field can reproduce the energetic cost of nucleosomal DNA unwinding,[S21–S23] the dependence of the unwinding barrier on applied tension,[S22] and the sequence-specific DNA binding strength to the histone octamer.[S24] The de Pablo group further showed that the model could reproduce the binding strength between a pair of nucleosomes measured in DNA origami-based force spectrometer experiments.[S25,S26]

The quantitative accuracy of the coarse-grained model in reproducing single nucleosome stability and inter-nucleosome interactions strongly supports its application to longer chromatin segments. Our prior study of tetranucleosomes further supports the model's accuracy in studying connected nucleosomes. For example, we simulated two di-nucleosomes with different link lengths and succeeded in resolving the structural difference, quantitatively reproducing FRET measurements from the van Noort group.[S27] More details about this comparison can be found in Figure S10 of Ref. S16. For the tetra-nucleosome, we predicted that stacked conformations with the two columns of nucleosomes more aligned have lower free energy than the PDB structure. This prediction was validated by independent simulations performed with an explicit solvent force field SIRAH[S28] and by all-atom simulations from the Wereszczynski group.[S29] More details about this comparison can be found in Figure S5 of Ref. S16.

Free Energy Profiles for Chromatin Under Tension

We defined two collective variables to explore chromatin configurations and compute free energy profiles. The unwrapping variable, q_{wrap}, quantifies DNA unwrapping using the distance between neighboring nucleosome $d_{i,i+1}$. It is defined as

$$q_{\text{wrap}} = \frac{1}{11}\sum_{i=1}^{11} \exp\left[-\frac{(\max(d_{i,i+1}, d_o) - d_o)^2}{2\sigma_w^2}\right]. \tag{S1}$$

$d_o = 15$ nm is close to the distance between two neighboring nucleosomes in the PDB structure (PDB ID: 1KX5),[S8] and we used $\sigma_w = 4$ nm. The function max selects the larger value of the two distances. The above definition makes use of the geometric constraint that increase in the distances between neighboring nucleosomes ($d_{i,i+1}$) can only arise from nucleosome unwrapping. The unstacking variable, d_{stack}, measures the mean distance between

i-th and $(i+2)$-th nucleosomes as

$$d_{\text{stack}} = \frac{1}{10}\sum_{i=1}^{10} d_{i,i+2}. \tag{S2}$$

Umbrella simulations with the two collective variables at forces 0-3 pN were carried out to compute the free energy profiles. To compare our simulations with force-extension experiments, we applied force f_{ext} along the z-axis projection of the DNA end-to-end distance (L_z). The two DNA ends were defined as the geometric centers of all the coarse-grained beads for the first and last five base pairs. The potential energy function of these simulations at center (q_o, d_o) and force f_{ext} is defined as

$$U_{\text{biased}} = U(\mathbf{r}) + \frac{\kappa_q}{2}(q_{\text{wrap}} - q_o)^2 + \frac{\kappa_d}{2}(d_{\text{stack}} - d_o)^2 - f_{\text{ext}}L_z, \tag{S3}$$

where $U(\mathbf{r})$ corresponds to the interaction energy defined by the force field. The umbrella centers (q_o, d_o) were initially placed on a uniform grid [0.45:0.90:0.15]×[10:30:5] nm. We introduced additional centers to improve the overlap between umbrella simulations. A complete list of the umbrella centers and the restraining constants is provided in Table S1.

At the extension force larger than 3 pN, the 12mer can adopt configurations that cover a wide range of q_{wrap} and d_{stack}. Uniform sampling of the entire accessible phase space becomes too costly computationally. Therefore, we only carried out one-dimensional umbrella simulations with d_{stack} as the collective variable. The corresponding potential energy function was defined as

$$U_{\text{biased}} = U(\mathbf{r}) + \frac{\kappa_d}{2}(d_{\text{stack}} - d_o)^2 - f_{\text{ext}}L_z. \tag{S4}$$

We used $\kappa_d = 0.05$ kcal/(mol · nm^2), and d_o spans from 10 to 50 nm with a step size of 2.5 nm.

Initial configurations from the neural network model

Conformational sampling of the coarse-grained model is challenging due to strong but non-specific electrostatic interactions. We initialized the umbrella sampling simulations with the most probable configurations predicted by a neural network under a similar setup to alleviate the sampling challenge. As detailed in the *Section: Neural Network Model for the 12mer Chromatin*, the neural network model quantifies the stability of chromatin configurations using inter-nucleosome distances. It is computationally efficient and allows equilibrium sampling of chromatin configurations.

For a coarse-grained umbrella simulation centered at (q_o^N, d_o^N) with extension force $f_{\text{ext}} \leq$ 3 pN, we carried out replica exchange Monte Carlo sampling of the following biased free energy

$$F_{\text{biased}} = F_{12}(\mathbf{d}) + \frac{\kappa_q}{2}(q_{\text{wrap}} - q_o^N)^2 + \frac{\kappa_d}{2}(d_{\text{stack}} - d_o^N)^2 - f_{\text{ext}}L, \tag{S5}$$

with $\kappa_q = 47.8$ kcal/mol and $\kappa_d = 4.78 \times 10^{-2}$ kcal/(mol $\cdot$ nm^2). $F_{12}(\mathbf{d})$ quantifies the free energy of the 12-mer as function of inter-nucleosome distances, $\mathbf{d}$, with a neural network model. More details about the free energy function can be found in the *Section: Neural Network Model for the 12mer Chromatin.* L is the distance between the first and the last nucleosomes. See *Section: Numerical Simulations of the Neural Network Model* for sampling details. We used the samples collected in the final 300000 steps of the 300K replica for a K-means clustering analysis with 10 centers. The configuration closest to the center of the largest cluster was selected as the most probable configuration.

For simulations with 3.5 pN force, we only performed umbrella sampling of the neural network model at a limited set of values for d_o^N = 10.0 nm, 15.0 nm, 20.0 nm, 25.0 nm, and 30.0 nm. q_o^N was set as 0.45. A total of five neural network configurations were constructed. We assigned these configurations to initialize coarse-grained simulations by minimizing the difference between d_o^N and the corresponding umbrella center of coarse-grained simulations.

For simulations with 4 pN force, we performed two sets of coarse-grained simulations.

These simulations were initialized with neural network configurations obtained from umbrella sampling with centers located at [q_o^N=0.45, 0.6] × [d_o^N=10:30:5] nm.

The neural network model represents chromatin structures with inter-nucleosome distances. We performed short targeted molecular dynamics simulations starting from the two-helix fiber to build coarse-grained model structures consistent with the most probable configurations from the neural network sampling. These simulations bias on all the inter-nucleosome distances with a restraining constant of 23.9 $\text{kcal}/(\text{mol} \cdot \text{nm}^2)$ for approximately 300000 steps. The end configurations of these simulations were used to initialize the coarse-grained umbrella simulations.

One-dimensional free energy calculations at 4.5 pN force

For simulations with 4.5 pN force, the neural network model is no longer sufficient for producing equilibrated, most probable starting configurations. It was trained using tetra-nucleosome configurations with a maximum extension of 50 nm, so the model can at most predict an end-to-end distance of 183 nm. This value is smaller than that anticipated in the linear regime ($\sim$ 270 nm). We performed two independent sets of umbrella simulations using different initial configurations as detailed below.

In the first set of simulations, we initialized the trajectories using the end configurations from the second set of 4 pN simulations presented in the main text (Table S1). A total of 17 simulations were performed.

From the first set of umbrella simulations, we observed that chromatin at large end-to-end distances tends to fall into configurations with clusters formed by neighboring nucleosomes. To sample more extended configurations, we introduced another collective variable, $d_{i,i+1}^{\min}$, defined as $\min(d_{i,i+1})$, to initialize the second set of umbrella simulations from the most probable configuration predicted from the neuronal network model at 4 pN. The new variable quantifies the minimal distance between nearest-neighbor nucleosomes. Explicit biases on $d_{i,i+1}^{\min}$ help overcome the energetic barrier associated with breaking these clusters. Specifically,

we ran nine umbrella-sampling simulations using harmonic biases on $d^{\min}_{i,i+1}$, with umbrella centers placed on a uniform grid of [5.0:25:2.5] nm and an umbrella bias of 0.0005 kcal/(mol$\cdot$nm^2). Each simulation lasted for 12.75 million steps and was performed with the presence of a 4.5 pN force on the DNA end-to-end distance. The end structures of these simulations were used to initialize production simulations at the 4.5 pN force with harmonic biases on d_{stack} as all simulations presented in the main text. The production runs lasted 25 million steps.

We combined the two data sets to estimate the chromatin extension at 4.5 pN.

Estimating the extension per nucleosome from experimental data

We processed the force-extension curve from single-molecule force spectroscopy experiments to compute the extension per nucleosome. The extension length from experiments includes contributions from the DNA handle and the chromatin. Following previous study,[S30] we estimate the DNA handle extension as

$$L_{z,\text{handle}} = L_{\text{c,handle}} \times \left(1 - \frac{1}{2}\sqrt{\frac{k_B T}{f_{\text{ext}} A}} + \frac{f_{\text{ext}}}{S}\right) \tag{S6}$$

where k_B is Boltzmann constant, T is temperature, A is the persistence length of DNA, f_{ext} is the extension force along the z-axis, and S is the stretching modulus. We used $A = 50$ nm, $S = 900$ pN, and $T = 300$ K. The contour length of the DNA handle, $L_{c,\text{handle}}$, is estimated as

$$L_{c,\text{handle}} = [n_{\text{bp}} - \text{NRL} \times (n_{\text{nucl}} - 1) - 147]b \tag{S7}$$

where n_{bp} is the total number of base pairs in DNA, NRL is nucleosomal repeat length, n_{nucl} is the total number of nucleosomes, and b is the length of each base pair. We used NRL = 167 bp, $n_{\text{nucl}} = 25$, $n_{\text{bp}} = 7045$ bp, and $b = 0.34$ nm.

Subtracting the extension of the DNA handle from the total extension length L_z, the

extension per nucleosome can be estimated as

$$L_{z,\text{nucl}} = \frac{L_z - L_{z,\text{handle}}}{n_{\text{nucl}} - 1}. \tag{S8}$$

Theoretical predictions of chromatin extension along the z-axis

To better understand the linear extension of chromatin at small forces, we introduced an analytical model based on simulation results without extension force.

We approximate the unbiased free energy profile for chromatin extension at zero force with a harmonic function, $F(L) = a(L - L_0)^2 + b$, where L is the extension length, i.e., the end-to-end distance. The parameters were obtained by a least-squares fitting to the simulation data presented in Figure 1C of the main text, resulting in $a = 1.200 \times 10^{-2}\ k_BT/\text{nm}^2$, $L_0 = 26.83$ nm, and $b = 0.3820\ k_BT$. The corresponding free energy profile with an extension force f along the z-axis can be defined as $F_f(L) = F(L) - fL\cos\theta$, where θ is the azimuthal angle (i.e. the angle between the fiber end-to-end distance direction and the z-axis). From this expression, the average extension along the z-axis can be computed as

$$\langle L_z \rangle_f = \frac{\int_0^\infty \int_0^\pi L\cos\theta e^{-\beta F_f(L)} L^2 dL \sin\theta d\theta}{\int_0^\infty \int_0^\pi e^{-\beta F_f(L)} L^2 dL \sin\theta d\theta} \tag{S9}$$

Numerical integration of the above equation led to $\langle L_z \rangle_f = 0$, 33.87, 45.76, and 56.36 nm for extension force of 0, 1, 2, and 3 pN, respectively. The extension per nucleosome along the z-axis (Z_{ext} per nucleosome) is defined as $\langle L_z \rangle_f/11$ and shown in Figure S7.

Decomposing Inter-nucleosome Distances into Shear and Normal Motions

As discussed in the main text, two distinct motions can increase the distance between i-th and $(i+2)$-th nucleosomes and the collective variable d_{stack}. To characterize these two motions quantitatively, we introduced a coordinate system for each nucleosome. Following de

Pablo and coworkers,[S25] we defined the origin of the coordinate system using the geometric center of residues 63-120, 165-217, 263-324, 398-462, 550-607, 652-704, 750-811, and 885-949. The IDs continuously index residues from chain A to chain H of PDB 1KX5. Two additional points were introduced to define the nucleosomal plane using the geometric center of the dyad that includes CG atoms 81-131, 568-618, and the geometric center of CG atoms 63-120, 165-217, 750-811, and 885-949. Two unit vectors, $\mathbf{u}$ and $\mathbf{v}$, can then be defined using the vectors pointing from the origin to the dyad and the third point. Atoms in the third point were chosen such that $\mathbf{u}$ and $\mathbf{v}$ are approximately orthogonal to each other. The unit normal vector $\mathbf{w}$ for nucleosome plane can then be defined as parallel to the cross product, $\mathbf{u} \times \mathbf{v}$. An illustration of the various axes is provided in Figure S11.

With the nucleosomal axes defined above, the distances between two nucleosomes can be decomposed to the distances within the nucleosomal plane, i.e., shearing, and the distance perpendicular to the plane, i.e., unstacking. Denoting the vector from nucleosome i to nucleosome $i+2$ as $\mathbf{d}_{i,i+2}$ (here we use the distance between the coordinate origins for the two nucleosomes), the corresponding normal and shear distances are $d^n_{i,i+2} = |\mathbf{d}_{i,i+2} \cdot \mathbf{w}_i|$ and $d^s_{i,i+2} = \sqrt{|\mathbf{d}_{i,i+2}|^2 - {d^n_{i,i+2}}^2}$. The normal and shear distances for the 12mer chromatin, d_n and d_s, are defined using the mean values of all nucleosome i and $i+2$ pairs as $d_n = \frac{1}{10}\sum_{i=1}^{10} d^n_{i,i+2}$ and $d_s = \frac{1}{10}\sum_{i=1}^{10} d^s_{i,i+2}$.

Free Energy Calculations for Two Interacting 12mers

To quantify the impact of chromatin-chromatin interactions and crowding on the stability of fibril configurations, we carried out simulations with two chromatin segments. Umbrella sampling was performed using two collective variables. The first variable quantifies the average extension of the two 12mers with $\bar{d}_{\text{stack}}$ defined as

$$\bar{d}_{\text{stack}} = \frac{1}{2}(d^1_{\text{stack}} + d^2_{\text{stack}}). \tag{S10}$$

d_{stack} is defined in Eq. S2 and $1, 2$ index the two 12mers. The second variable measures the number of contacts between the two chromatins. Contacts were defined at the nucleosome level, and a pair of nucleosomes is denoted as in-contact if the distance between their geometric centers ($d_{i,j}$) is less than 15 nm. Mathematically, the interchain contacts, C, is defined as

$$C = \sum_{i=1}^{12} \sum_{j=13}^{24} \frac{1 - (d_{i,j}/d_o)^6}{1 - (d_{i,j}/d_o)^{12}}, \tag{S11}$$

where i and j indices over nucleosomes from the two 12mers and $d_o = 15$ nm.

We biased the simulations towards various collective variable values for a comprehensive exploration of the phase space. Details of the umbrella centers and force restraints used in our simulations are provided in Table S2. Simulations with umbrella centers $\bar{d}_{\text{stack}}$ biased to values $\leq$ 10nm were initialized using the two-helix fibril configuration for each chromatin placed at $\geq$ 20 nm apart. The rest of the simulations were initialized with extended chromatin configurations extracted from the neural network simulations. Chromatin configurations in simulations with 4 pN extension force at umbrella centers d_o were adopted here for simulations that biased $\bar{d}_{\text{stack}}$ to the same values. Only chromatin configurations for the first set of simulations with 4 pN extension force were used here (see *Section: One-dimensional free energy calculations at 4 pN*). The initial five million steps of each trajectory were discarded as equilibration in our free energy calculations.

Neural Network Model for the 12mer Chromatin

To facilitate conformational sampling of the 12mer, we introduced a neural network model to quantify the free energy of chromatin configurations as a function of inter-nucleosomal distances. The neural network model is a generalization of the free energy surface for a tetra-nucleosome determined in a previous study.[S16]

Parameterizing the Tetra-nucleosome Free Energy Landscape with Neural Networks

In Ref. S16, we parameterized a neural network model to compute the free energy of a tetra-nucleosome (A) from the six internuclesome distances ($\mathbf{d}$).

To make the neural network's output invariant with respect to nucleosome indexing order, i.e., $A(\mathbf{d} = (d_{12}, d_{13}, d_{14}, d_{23}, d_{24}, d_{34})) = A(\tilde{\mathbf{d}} = (d_{34}, d_{24}, d_{14}, d_{23}, d_{13}, d_{12}))$, we further converted the inter-nucleosome distances into symmetrical features $\mathbf{s}(\mathbf{d}) = (s_1(\mathbf{d}),\ s_2(\mathbf{d}),\ s_3(\mathbf{d}),\ s_4(\mathbf{d}),\ s_5(\mathbf{d}),\ s_6(\mathbf{d}))$ as follows:

$$
\begin{aligned}
s_1 &= d_{12} + d_{34} \\
s_2 &= d_{13} + d_{24} \\
s_3 &= d_{14} \\
s_4 &= d_{23} \\
s_5 &= d_{12} \cdot d_{13} + d_{24} \cdot d_{34} \\
s_6 &= d_{12} \cdot d_{13}^2 + d_{24}^2 \cdot d_{34}.
\end{aligned}
\tag{S12}
$$

From the above definition, it is straightforward to verify that $\mathbf{s}(\mathbf{d}) = \mathbf{s}(\tilde{\mathbf{d}})$. In addition, given any $\mathbf{s}$ in the range of $\mathbf{s}(\mathbf{d})$, two solutions of $\mathbf{d}$ exist for Eq. (S12) and these two solutions corresponds to the two different ways of indexing nucleosome. Specifically, if one of the solution is $\mathbf{d} = (d_{12}, d_{13}, d_{14}, d_{23}, d_{24}, d_{34})$, the other solution will be $\tilde{\mathbf{d}} = (d_{34}, d_{24}, d_{14}, d_{23}, d_{13}, d_{12})$. Therefore, the features $\mathbf{s}(\mathbf{d})$ are symmetric and only symmetric to the two ways of indexing nucleosomes.

Using the symmetric features as input, i.e., $A(\mathbf{d}) = A(\mathbf{s}(\mathbf{d}))$, a neural network with two fully connected hidden layers, each of which has 200 nodes, was used to parameterize the free energy. The neural network was trained by minimizing the loss function

$$
||(-\nabla A(\mathbf{d})) - \mathbf{F}(\mathbf{d})||^2 + \lambda||\mathbf{w}||^2, \tag{S13}
$$

where $\mathbf{w}$ are weight parameters of the neural network. $\mathbf{F}(\mathbf{d})$ are mean forces at $\mathbf{d}$ estimated using restrained molecular dynamics simulations (see below). $\lambda = 6 \times 10^{-4}$ is the weight decay factor and acts as a regularizer of optimization. Overall, the neural network has 41801 ($7 \times 200 + 201 \times 200 + 201 \times 1 = 41801$) parameters, which is smaller than the total number of constraints $10000 \times 6 = 60000$. The Adam optimizer[S31] was used to train the neural network for 100000 steps with a learning rate of 0.001. To prevent over fitting and improve the robustness of neural networks, we trained 30 models independently and used the average results to estimate the final free energy.

To estimate mean forces at different chromatin configurations with inter-nucleosome distances $\mathbf{d_o}$, we carried out restrained molecular dynamics simulations with the harmonic biasing potential

$$V_b = \frac{1}{2}\sum_{i=1}^{3}\sum_{j=i+1}^{4} k(d_{ij}(\mathbf{r}) - d_{ij}^{o})^2, \tag{S14}$$

where i and j are indexes of the four nucleosomes, $k = 1000$ kJ/(mol·nm^2), and d_{ij}^{o} is the inter-nucleosome distances between nucleosome i and j for the selected center $\mathbf{d_o}$. The mean forces were estimated as

$$\mathbf{F}_{ij}^{o} = \frac{1}{T}\sum_{t=1}^{T} k(d_{ij}^{t} - d_{ij}^{o}). \tag{S15}$$

Here $T = 50,000$ represents the number of configurations collected from a 500,000 step-long trajectory.

Ten thousand tetra-nucleosome configurations were selected to compute mean forces and parameterize the neural network. To ensure that these configurations cover relevant structures for chromatin folding, we selected them from simulation trajectories that repeatedly probe chromatin folding and unfolding. These trajectories were performed by combining metadynamics with temperature accelerated molecular dynamics (TAMD) to bias the simulations along two collective variables R_g and Q. The radius of gyration, R_g, is defined

as

$$R_g = \sqrt{\frac{1}{4}\sum_{i=1}^{4}(\mathbf{r}_i - \mathbf{r}_{\text{com}})^2}, \tag{S16}$$

where $\mathbf{r}_i$ is the geometric center of the i-th nucleosome using the coordinates of nucleosome core histone residues. $\mathbf{r}_{\text{com}}$ is the center of mass coordinate for all nucleosomes. Q measures the similarity of a given tetra-nuclesome configuration to the crystal structure (PDB ID: 1ZBB) and is defined as

$$Q = \frac{1}{6}\sum_{i=1}^{3}\sum_{j=i+1}^{4}\exp\left[-\frac{(r_{ij} - r_{ij}^o)^2}{2\sigma^2}\right], \tag{S17}$$

where r_{ij} measures the distance between the center of the two nucleosomes. More simulation details can be found in Ref. S16.

Generalizing Tetra-nucleosome Results to 12mer Chromatin

We generalized the tetra-nucleosome neural network model to estimate the free energy of 12mer. We defined $F_n(1\ldots,n)$ as the free energy of an oligomer including n nucleosomes of indices $1,\ldots,n$. We assumed that each nucleosome with index i could only interact with nucleosomes $i \pm 1/2/3$, ignoring nucleosome pair interactions beyond tetramers. As shown in Figure S4, mean distances for these nucleosome pairs are much larger and no interactions are expected among them. Under this assumption, the free energy of $n+1$ nucleosomes (F_{n+1}) can be determined from the following recursive relationship as

$$F_{n+1}(1,\ldots,n+1) = F_n(1,\ldots,n) + F_4(n-2,n-1,n,n+1) - F_3(n-2,n-1,n). \tag{S18}$$

Subtracting the free energy (F_3) avoids the double-counting from adding the tetrameric contribution (F_4).

The trimer free energy is estimated as follows. Assuming that the fourth nucleosome is far away from the rest of the three and its interaction with them can be ignored, the free

energy difference between the two should be a constant. Therefore, we have

$$F_3(d_{1,2}, d_{1,3}, d_{2,3}) = F_4(d_{1,2}, d_{1,3}, d_{2,3}, d_{1,4} = d_{2,4} = d_{3,4} = 15\text{nm}) + \text{const.} \tag{S19}$$

Here $d_{i,j}$ refers the distance between nucleosome i and j. The distances from the fourth nucleosome to the other three (i.e. $d_{1,4}, d_{2,4}, d_{3,4}$) were set as 15 nm. For $d_{3,4}$, this value is comparable to the distance between neighboring nucleosomes in the PDB structure for a tetra-nucleosome to avoid significant DNA unwrapping or DNA overstretching. It is also large enough to unstack i and $i \pm 2$ nucleosomes and to dissociate i and $i \pm 3$ nucleosome contacts, based on previous computational results.[S25] The effectiveness of this generalized neural network model is verified based on the fact that it can accurately predict the extension at different forces (Figure S5).

Given all the $d_{i,i\pm1/2/3}$ and assuming a left-handed helix, the relative position of each nucleosome can be uniquely determined geometrically, as long as the distances satisfy some geometric requirements such as triangle inequality. After determining the relative position of each nucleosome, the full set of distances (i.e. distances between any two different nucleosomes) was used to bias coarse-grained simulations towards the most probable configurations predicted by the neural network sampling.

Numerical Simulations of the Neural Network Model

We used the replica-exchange Monte Carlo algorithm to explore the free energy surface defined by the neural network. 20 Replicas with temperatures as the geometric sequence from 300 K to 2000 K were used. 500000 steps of simulations were performed for each replica. The initial 20000 steps were used to optimize the MC simulation step size so that the mean acceptance rate of MC movement is $\sim$ 0.20-0.25. The exchange between two neighboring replicas was attempted every 50 steps. We used the samples collected in the final 300000 steps of the replica at 300 K for analysis.

Details of Validation Simulations

Simulations Starting from Uniformly Extended Chromatin Configurations

As detailed in the *Section: Free Energy Profiles for Chromatin Under Tension*, we used chromatin configurations predicted by the neural network model to initialize the umbrella simulations. From the initial configurations, we performed extensive, long molecular dynamics simulations to alleviate any biases that the neural network model might have introduced.

We acknowledge that, despite our best effort, it remains possible that the simulations are not sufficient to remove biases that the neural network might introduce. As an additional test, we carried out a new set of umbrella simulations starting from uniformly extended chromatin structures. By design, the initial configurations are free of clutches. These simulations were again performed with the presence of a 4 pN extension force for direct comparison with results presented in the main text.

To facilitate the conformational sampling of clutched versus uniform chromatin conformations, we performed two dimensional umbrella simulations using both d_{stack} and α. As mentioned in the main text, d_{stack} uses the average distance between 1-3 nucleosomes to measure the average chromatin extension. α is defined as the ratio of the maximum and minimum distance between 1-3 nucleosomes, i.e., $\alpha = d_{i,i+2}^{\text{max}}/d_{i,i+2}^{\text{min}}$. For clutched configurations, the distance at the interface between two nucleosome clusters is expected to be much larger than the distance between nucleosomes within the same cluster, and α will be much larger than one. On the other hand, for more uniformly extended configurations, α will approach one. The maximal and minimal values of $d_{i,i+2}$ were computed with the following

expressions with analytical derivatives

$$
\begin{aligned}
d_{i,i+2}^{\max} =& \beta_1 \ln\left(\sum_{i=1}^{10} e^{d_{i,i+2}/\beta_1}\right) \\
d_{i,i+2}^{\min} =& \beta_2 / \ln\left(\sum_{i=1}^{10} e^{\beta_2/d_{i,i+2}}\right),
\end{aligned}
\tag{S20}
$$

where $\beta_1 = 0.1$ nm and $\beta_2 = 1000$ nm. The list of umbrella centers is provided in Table S3.

We initialized these umbrella simulations with two uniform chromatin configurations that lack nucleosome clutches (Figure S13A). Both configurations were obtained from biased simulations initialized with the fibril structures. The less extended uniform structure with an end-to-end distance per nucleosome of 7.74 nm was produced by restricting pair-wise nucleosome distances $d_{i,i+1}, d_{i,i+2}, d_{i,i+3}$ to 15, 20, 25 nm, respectively. The more extended uniform chromatin structure with an end-to-end distance per nucleosome of 13.64 nm was prepared with a constant-velocity pulling simulation that stretches the end-to-end distance to 150 nm. The entire 147 bp nucleosomal DNA and the histone core proteins were rigidified during the biasing simulations to prevent DNA unwrapping and clutch formation.

From the two initial configurations, we first carried out 0.5 million steps equilibration simulations to relax them towards individual umbrella centers. Simulations with $d_{\text{stack}} \leq 20$ nm started from the structure with a end-to-end distance per nucleosome of 7.74 nm (Figure S13A, top), and the rest of the simulations started from the second structure. The relaxation was achieved with a moving harmonic restraint $U_{\text{relax}}(t)$ defined as

$$
U_{\text{relax}}(t) = \frac{1}{2}\left[\kappa_{d_{\text{stack}}}(t)(d_{\text{stack}} - d_{\text{stack},0}(t))^2 + \kappa_\alpha(t)(\alpha - \alpha_0(t))^2\right], \tag{S21}
$$

where $\kappa_{d_{\text{stack}}}(t)$ and $\kappa_\alpha(t)$ are time-dependent harmonic restraint constants. $d_{\text{stack},0}(t)$ and $\alpha_0(t)$ are time-dependent moving restraint centers. Values for these time-dependent quantities are provided in Table S4.

During the relaxation period, for umbrella centers with $d_{\text{stack}} \leq 25$ nm and $\alpha \leq 4$, we kept

the entire 147 bp nucleosomal DNA and the histone core rigidified to avoid DNA unwrapping and clutch formation. For umbrella simulations at larger d_{stack} values, no such restrictions were applied since doing so may prevent chromatin extension.

After equilibration, we launched production simulations that lasted 10 million steps and saved the configurations every 5000 steps. The production simulations used the same force field setup as those presented in the main text. The first three million steps were discarded and the rest of the data were used for free energy calculations.

Simulations with Fully Rigidified Nucleosomes

To explore the role of DNA unwrapping on nucleosome clutch formation and inter-chain contacts, we performed additional simulations with fully rigidified nucleosomes. Unlike simulations presented in the main text, the entire 147 bp nucleosomal DNA and the histone core were constrained together as rigid bodies in the native configurations using the same algorithms. Only linker DNA and histone tails remain flexible.

Chromatin extension under 4 pN force

To more directly evaluate the impact of DNA unwrapping on clutch formation, we carried out a new set of simulations with fully rigidified nucleosomes to study chromatin extension under 4 pN force. A total of 10 umbrella simulations were performed to bias d_{stack} to values between 10 nm to 32.5 nm, with an increment of 2.5 nm. We set the umbrella restraining constant $\kappa = 100.0$ kcal/(mol $\cdot$ nm^2) in the first 400,000 steps to drive chromatin configurations towards the restraining centers. After that, the umbrella bias was relaxed to $\kappa = 0.05$ kcal/(mol $\cdot$ nm^2) and the simulations continued for another 15 millions steps. The initial 1 million steps were excluded when calculating the free energy profile. Umbrella centers and force restraints used in these simulations are provided in Table S5.

Two sets of configurations were used to initialize the above simulations. They were produced by constant-velocity pulling simulations over 5 million steps initialized from a

fibril structure. The pulling bias was applied to the z-axis projection of the end-to-end distance. Fiver pulling simulations were performed using independent random seeds with a target bias of 75 nm, and another five with a target bias of 150 nm. In total, these pulling simulations produced ten configurations. The first five 75 nm configurations were used to initialize umbrella simulations centered between 10 nm to 20 nm. The second five 150 nm configurations were used to initialize umbrella simulations centered between 22.5 nm to 32.5 nm.

Inter-chain contacts with two 12mer simulations

To explore the contribution of nucleosomal DNA unwrapping to inter-chain contacts, we carried out additional simulations following the same protocol as that described in *Section: Free Energy Calculations for Two Interacting 12mers*, but with fully rigidified nucleosomes. Initial configurations of these simulations were obtained from a constant-velocity pulling simulation that drives the chromatin z-axis extension towards 75 nm over 20 million steps. The two 12mers adopt identical configurations at the beginning of the simulations and were separated 20 nm part as measured by the center-of-mass distance. Umbrella centers and harmonic restraining constants used in these simulations are provided in Table S5. The initial 5 million steps of the umbrella sampling were discarded as equilibration.

Table S1: Summary of umbrella simulation details for free energy calculations at various extension forces. The format for umbrella centers, "start:end:step", indicates the a series of values from "start" to "end" with a spacing of "step". The two restraining constants are shown in the format "($\kappa_{q_{\mathrm{wrap}}}$ (kcal/mol), $\kappa_{d_{\mathrm{stack}}}$ (kcal/(mol $\cdot$ nm^2)))".

Extension force (pN)	Umbrella center: q_{wrap}	Umbrella center: d_{stack} (nm)	Restraining constants	Simulation length (million steps)
0	0.45:0.90:0.15	10.0:30.0:5.0	(50, 0.05)	10.5
0	1.00	6.0:10.0:0.5	(47.8, 1.20)	10
0	1.00	10.0:15.0:2.5	(47.8, 0.120)	10
0	1.00	12.5:15.0:2.5	(47.8, 0.478)	10
0	0.75:0.95:0.05	6.0:10.0:0.5	(120, 1.20)	10
0	0.90:0.95:0.05	10.0:15.0:2.5	(120, 0.120)	10
0	0.90:0.95:0.05	12.5:15.0:2.5	(120, 0.478)	10
0	0.80:0.85:0.05	10.0:20.0:2.5	(120, 0.0120)	10
0	0.75:0.85:0.05	12.5:20.0:2.5	(120, 0.478)	10
1	0.45:0.90:0.15	10.0:30.0:5.0	(50, 0.05)	10
2	0.45:0.90:0.15	10.0:30.0:5.0	(50, 0.05)	10
3	0.45:0.90:0.15	10.0:30.0:5.0	(50, 0.05)	15
3	0.45	10.0:20.0:5.0	(50, 0.2)	15
3	0.60	10.0:20.0:5.0	(50, 0.2)	15
3	0.75	10.0:30.0:5.0	(50, 0.2)	15
3	0.90	10.0:30.0:5.0	(50, 0.2)	15
3.5 (1st set)	n.a.	10.0:50.0:2.5	(0, 0.05)	25
4 (1st set)	n.a.	10.0:50.0:2.5	(0, 0.05)	24.5
4 (2nd set)	n.a.	10.0:50.0:2.5	(0, 0.05)	25
4.5 (1st set)	n.a.	10.0:50.0:2.5	(0, 0.05)	25
4.5 (2nd set)	n.a.	10.0:50.0:2.5	(0, 0.05)	25
4.5 (3rd set)	n.a.	47.5:50.0:2.5	(0, 0.5)	25

Table S2: Summary of umbrella simulation details for free energy calculations with two 12-mers. The same format as in Table S1 is adopted here. The two restraining constants are shown in the format "(κ_C (kcal/mol), $\kappa_{\bar{d}}$ (kcal/(mol $\cdot$ nm^2)))"

Umbrella center: C	Umbrella center: $\bar{d}$ (nm)	Restraining constants	Simulation length (million steps)
30.0:45.0:5.0	10.0:25.0:2.5	(0.1, 0.05)	20
10.0:20.0:5.0	6.0:10.0:0.5	(0.120, 1.20)	10
10.0:20.0:5.0	10.0:25.0:2.5	(0.478, 0.239)	10
10.0:20.0:5.0	10.0:25.0:2.5	(0.120, 0.0478)	10
10.0:20.0:5.0	9.0:9.5:0.5	(0.120, 4.78)	10
25.0:45.0:5.0	6.0:10.0:0.5	(0.120, 1.20)	20
25.0	10.0:25.0:2.5	(0.120, 0.0478)	20
25.0:45.0:5.0	9.0:9.5:0.5	(0.120, 4.78)	20
25.0	10.0	(0.120, 4.78)	20
25.0:45.0:5.0	10.0	(0.478, 0.239)	20
30.0:45.0:5.0	10.0	(0.120, 0.0478)	20
30.0:40.0:5.0	12.5:15.0:2.5	(0.478, 0.239)	20

Table S3: Summary of umbrella simulation details for free energy calculations using d_{stack} and α as collective variables. The same format as in Table S1 is adopted here. The two restraining constants are shown in the format "($\kappa_{d_{\text{stack}}}$ (kcal/(mol · nm^2)), κ_α (kcal/mol))"

Umbrella center: d_{stack} (nm)	Umbrella center: α	Restraining constants	Simulation length (million steps)
10.0:35.0:2.5	2.0:8.0:2.0	(0.05, 0.2)	10
22.5:27.5:2.5	2.0:8.0:2.0	(0.2, 0.2)	10
10.0:25.0:2.5	2.0:4.0:2.0	(0.05, 0.5)	10

Table S4: Summary of simulations with moving restraints to target chromatin configurations towards specific umbrella centers using d_{stack} and α as collective variables. The format for "restraining constants and centers" is ($\kappa_{d_{\text{stack}}}$ (kcal/(mol · nm^2)), κ_α (kcal/mol)), ($d_{\text{stack},0}$ (nm), α_0 (1)). We only listed the restraining constants and centers at simulation time of zero, 4×10^5 and 5×10^5 steps, and values in between these time points were updated via linear interpolation during the simulation.

Umbrella center: d_{stack} (nm)	Umbrella center: α	Restraining constants and centers at $t = 0$	Restraining constants and centers at $t = 4 \times 10^5$ steps	Restraining constants and centers at $t = 4 \times 10^5$ steps
10.0:25.0:2.5	2.0:4.0:2.0	(50, 200), (30, 1)	(50, 200), (d_{stack}, α)	(0.05, 0.2), (d_{stack}, α)
10.0:20.0:2.5	6.0:8.0:2.0	(50, 200), (20, 1)	(50, 200), (d_{stack}, α)	(0.05, 0.2), (d_{stack}, α)
22.5:25.0:2.5	6.0:8.0:2.0	(50, 200), (30, 1)	(50, 200), (d_{stack}, α)	(0.05, 0.2), (d_{stack}, α)
27.5:35.0:2.5	2.0:8.0:2.0	(50, 200), (30, 1)	(50, 200), (d_{stack}, α)	(0.05, 0.2), (d_{stack}, α)

Table S5: Summary of umbrella simulation details for free energy calculations with two 12-mers with fully rigidified nucleosomes. The same format as in Table S1 is adopted here, and the units for the two restraining constants are κ_C (kcal/mol), $\kappa_{\bar{d}_{\text{stack}}}$ (kcal/(mol $\cdot$ nm^2)).

Umbrella center: C	Umbrella center: $\bar{d}_{\text{stack}}$ (nm)	Restraining constants	Simulation length (million steps)
30.0:55.0:5.0	6.0:10.0:0.5	(0.0478, 0.478)	20
30.0:55.0:5.0	10.0:17.5:2.5	(0.0478, 0.239)	20

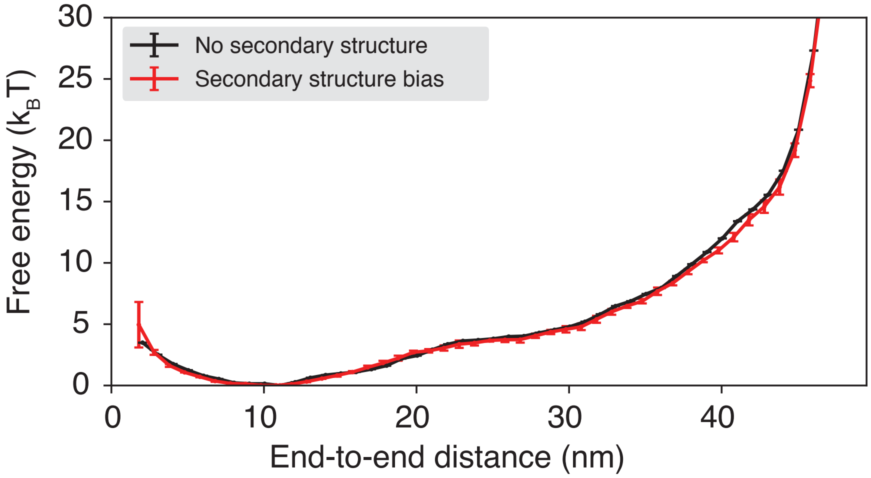

Figure S1: Secondary structure motifs for disordered histone tails negligibly impact nucleosome stability and protein-DNA interactions. The two curves correspond to the free energy profiles of the outer layer nucleosomal DNA unwrapping as a function of the DNA end-to-end distance. These profiles were determined from replica-exchange umbrella simulations with biases on the end-to-end distance of the nucleosomal DNA. The two sets of simulations only differ in the treatment of histone tails but otherwise share identical settings. The black curve was computed using simulations performed with the same model as that presented in the main text. On the other hand, the red curve was determined using simulations that explicitly accounted for secondary structure biases in the disordered histone tails. In particular, we used AlphaFold2[S32] to predict the structure of all the histone tails. We built new structure-based models for histone tails that account for the bonds, angles, and dihedrals from these initial structures. Therefore, the new models should reproduce the residue folding of histone tails and their tendency to form any secondary/tertiary structures. The umbrella centers were placed on a uniform grid [5.0:70.0:5.0] nm. The temperature replica exchange was applied between temperatures from 300 K to 410 K with a spacing of 10 K. Each simulation replica lasted for 5.5 million steps with a time step of 10.0 fs, and the first 250k steps were excluded for equilibration. We used the WHAM algorithm[S3] to process the simulation data from all temperatures and compute the free energy profiles. Error bars correspond to the standard deviation of the means estimated from three independent data blocks.

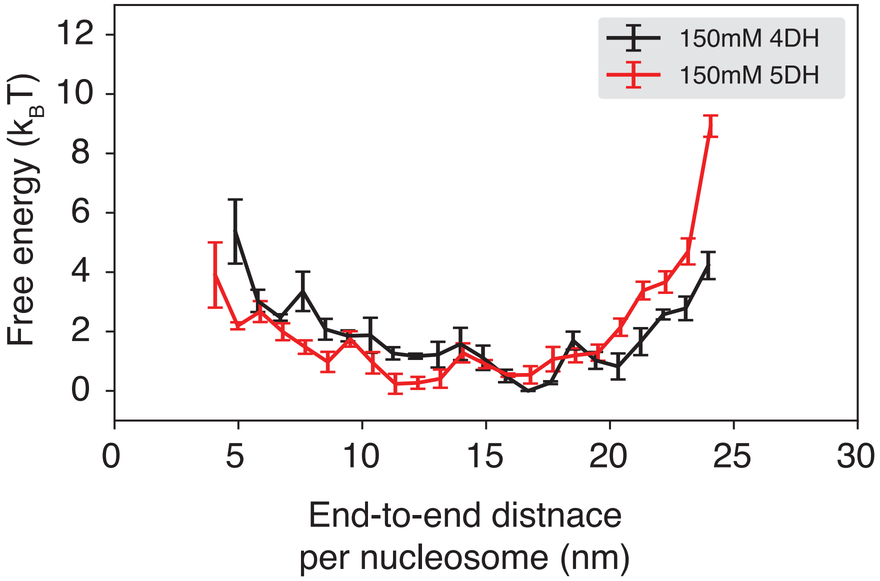

Figure S2: The cutoff distance used for the Debye Hückel potential has negligible impact on the computed free energy profile. The black line is identical to the one presented in Figure 1 of the main text. The red curve was computed with a new set of simulations that adopted a cutoff distance of five times Debye screening length. The new simulations were carried out following the same simulation protocol as those presented in the main text with the presence of 4 pN force.

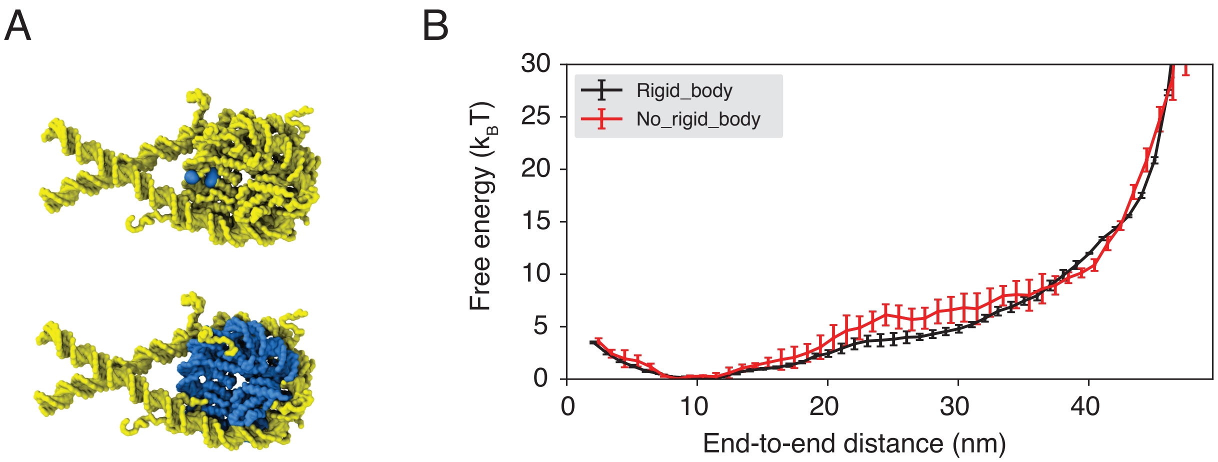

Figure S3: Rigidifying the inner layer nucleosomal DNA does not impact the energetics of outer layer DNA unwrapping. (A) Illustration of the groups of atoms rigidified in simulations. For simulations presented in the main text (bottom), both the histone core and inner layer (73 bp) of nucleosome DNA (shown in blue) are treated together as one rigid body. As an alternative treatment (top), we only rigidified the four residues and two nucleotides (shown in blue) located on the dyad axis to avoid nucleosomal DNA sliding. (B) Free energy profiles of outer layer nucleosomal DNA unwrapping as a function of the DNA end-to-end distance. These profiles were determined from replica-exchange umbrella simulations with biases on the end-to-end distance of the nucleosomal DNA. The two sets of simulations only differ in the treatment of rigid groups, as illustrated in part A, but otherwise share identical settings. The umbrella centers were placed on a uniform grid [5.0:70.0:5.0] nm. The temperature replica exchange was applied between temperatures from 300 K to 410 K with a spacing of 10 K. Exchanges among the replicas were attempted every 100 steps. Each simulation replica lasted for at least 5.5 million steps. The simulations that rigidified both the histone core and inner layer of nucleosomal DNA used a time step of 10.0 fs. The simulations that only rigidified the four residues and two nucleotides on the dyad axis require a smaller time step of 1.0 fs to ensure energy conservation. In both cases, the first 250k steps were excluded for equilibration. We used the WHAM algorithm[S3] to process the simulation data from all temperatures and compute the free energy profiles. Error bars correspond to the standard deviation of the means estimated from three independent data blocks.

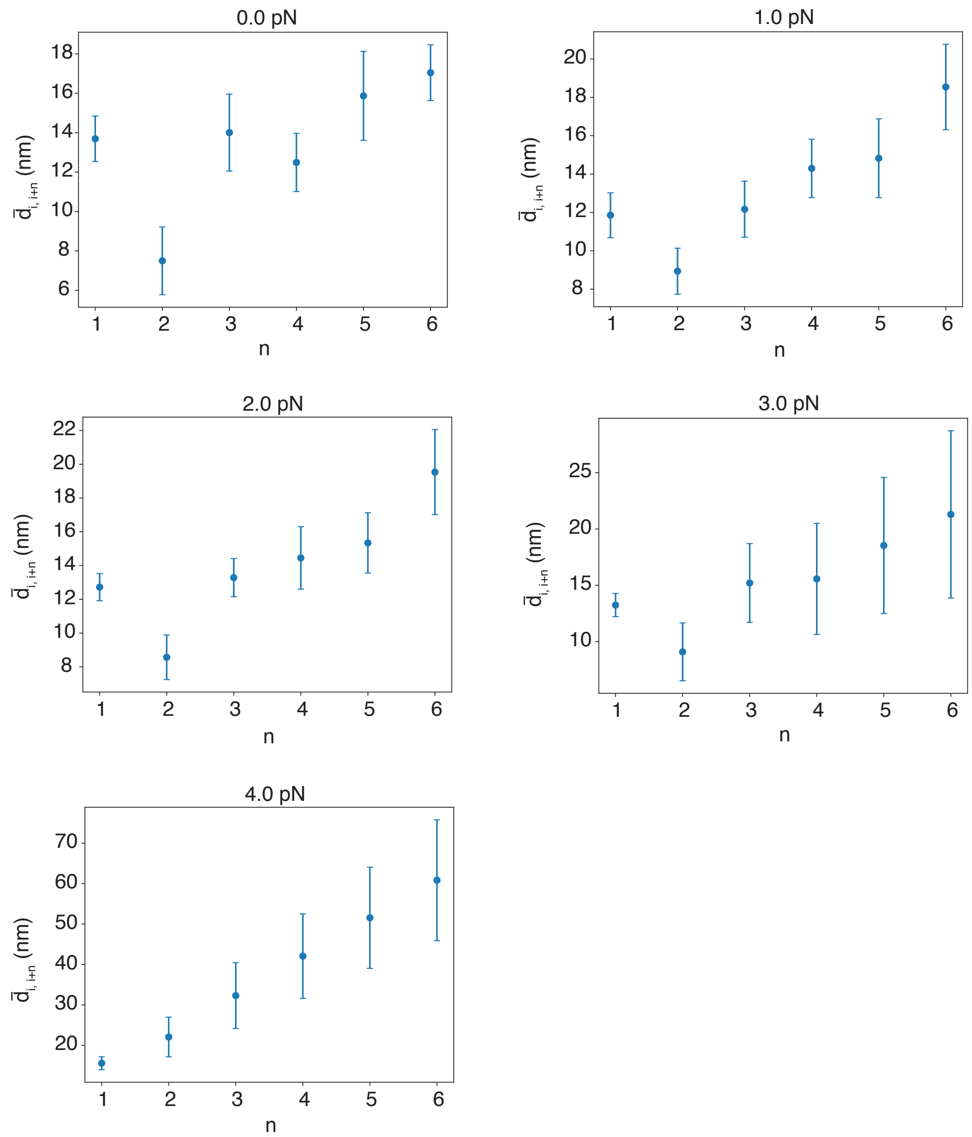

Figure S4: Mean distances between pairs of nucleosomes at various values of nucleosome separation n. Error bars correspond to the standard deviation of the mean estimated from three independent data blocks. These data suggest that the average distance between nucleosome pairs separated by four or more nucleosomes is larger than 13 nm. Therefore, nonbonded interactions between these nucleosomes contribute negligibly to the overall potential energy and stability of the chromatin structure. Therefore, neglecting their contribution to the chromatin conformational free energy in the neural network model is a reasonable approximation. See *Section: Neural Network Model for the 12mer Chromatin* for more details on the neural network model.

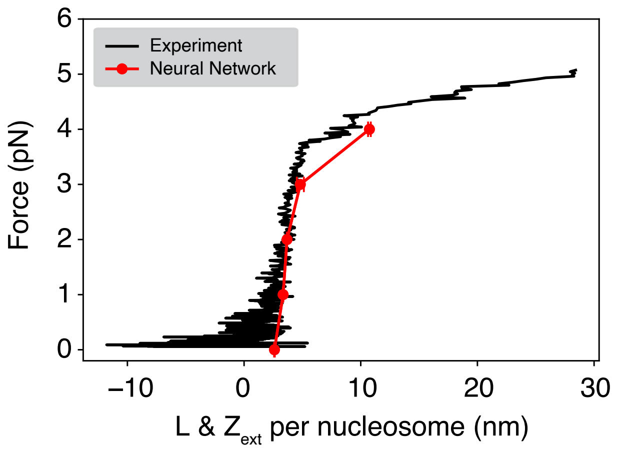

Figure S5: Comparison between experimental[S33] force-extension curve (black) and the one predicted by the neural network model. The neural network model quantifies chromatin stability as a function of inter-nucleosome distances. Based on the derivation shown in Eq. S9, when the extension force is larger than 1 pN, the extension along z-axis (L_z) is very close to the end-to-end distance (L), so that we approximated the z-axis extension per nucleosome using the distance between first and last nucleosome (L) divided by 11. L at different extension forces was calculated using umbrella simulations of the neural network model. See text *Section: Initial configurations from the neural network model* and *Section: Neural Network Model for the 12mer Chromatin* for simulation details.

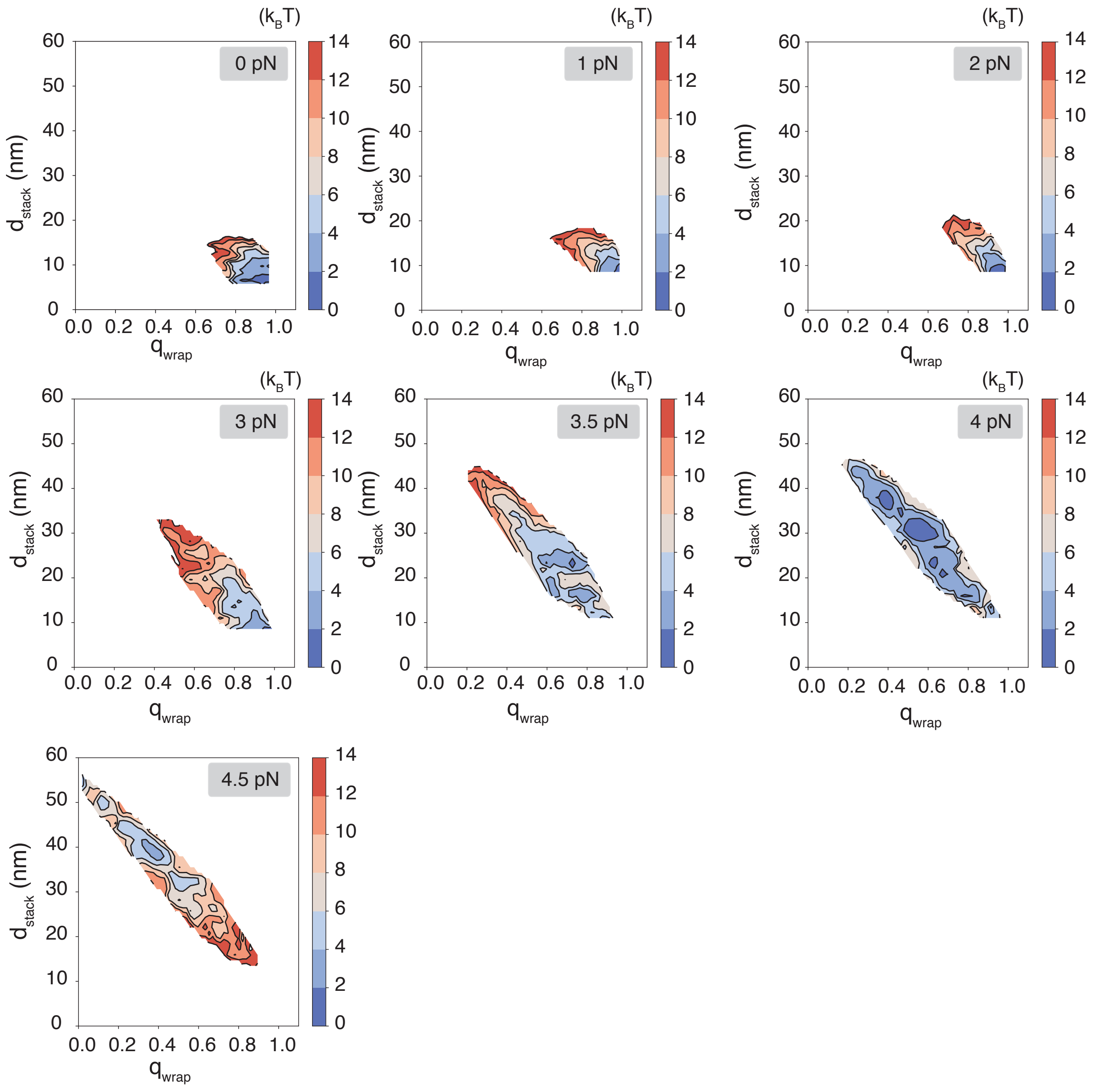

Figure S6: Two dimensional free energy profiles as a function of nucleosome unwrapping ($q_{\rm wrap}$) and unstacking ($d_{\rm stack}$) at various extension forces determined from umbrella simulations. See text *Section: Free Energy Profiles for Chromatin Under Tension* for simulation details.

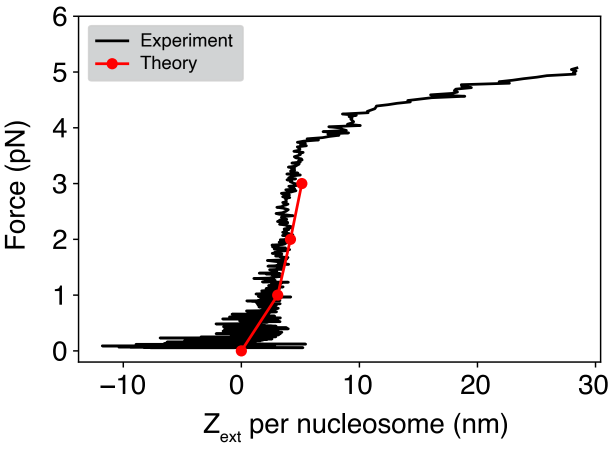

Figure S7: Theoretical predictions of chromatin extension along the z-axis, Z_{ext}. We assumed a harmonic potential for the end-to-end distance of the unbiased chromatin. Parameters in the potential were obtained from a least-square fitting to the simulation results shown in Figure 1C at 0 pN. From the harmonic potential, Z_{ext} can be computed with the analytical expression provided in Eq. S9. See *Section: Theoretical predictions of chromatin extension along the z-axis* for a detailed discussion.

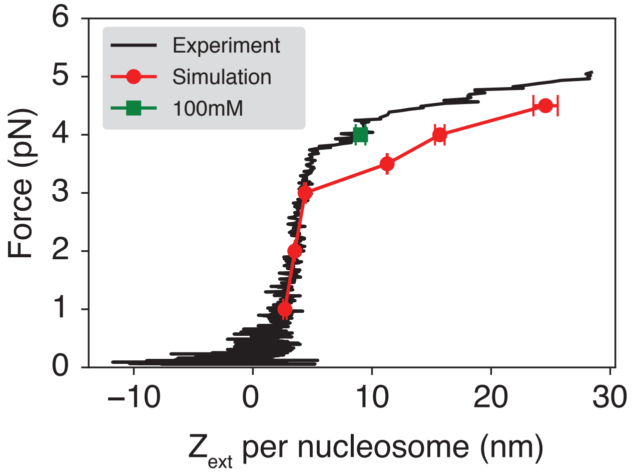

Figure S8: Comparison between the simulated (red) and experimental[S33] (black) force-extension curves. The results for simulations performed with 150 mM monovalent ions are reproduced from Fig. 1B. The green dot corresponds to chromatin extension at 4pN force obtained from simulations with 100 mM monovalent ions. We note that while previous experimental studies[S34] have shown that lower salt concentrations lead to chromatin decompaction, our results do not contradict them. A critical difference between the results presented here and previous experimental studies is the presence of force. In previous studies, chromatin was probed without any tension and should, in general, adopt compact conformations. For compact chromatin, linker DNAs come in close contact and contribute significantly to chromatin stability. Therefore, factors that affect their repulsion, such as increasing salt concentration, will dramatically impact chromatin extension. However, with 4 pN force, chromatin adopts much more extended configurations with very few contacts between linker DNA (Figure 2 of the main text). Histone-DNA interactions become more important for chromatin stability and extension in these configurations as many nucleosomes have unwrapped. Therefore, lowering the salt concentration would enhance attraction between histone proteins and DNA to stabilize individual nucleosomes and reduce chromatin extension. Consistent with this interpretation, many experimental studies have shown that nucleosome unwrapping becomes more prevalent at higher salt concentrations.[S35–S38]

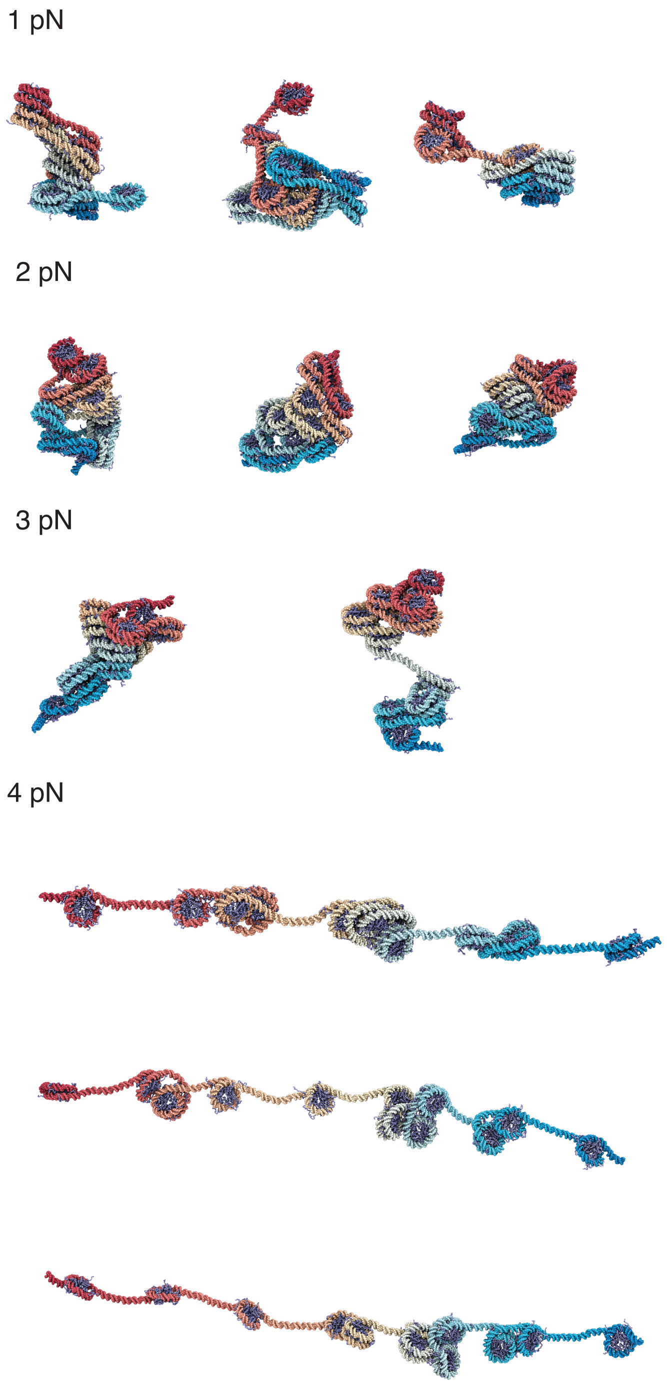

Figure S9: Additional representative chromatin structures from simulations performed under various extension forces. The values for the extension force are provided next to the structures. Similar to the ones shown in Figure 2 of the main text, these structures correspond to the central configurations of the clusters identified by the single-linkage algorithm using root mean squared distance (RMSD) as the distance between structures.

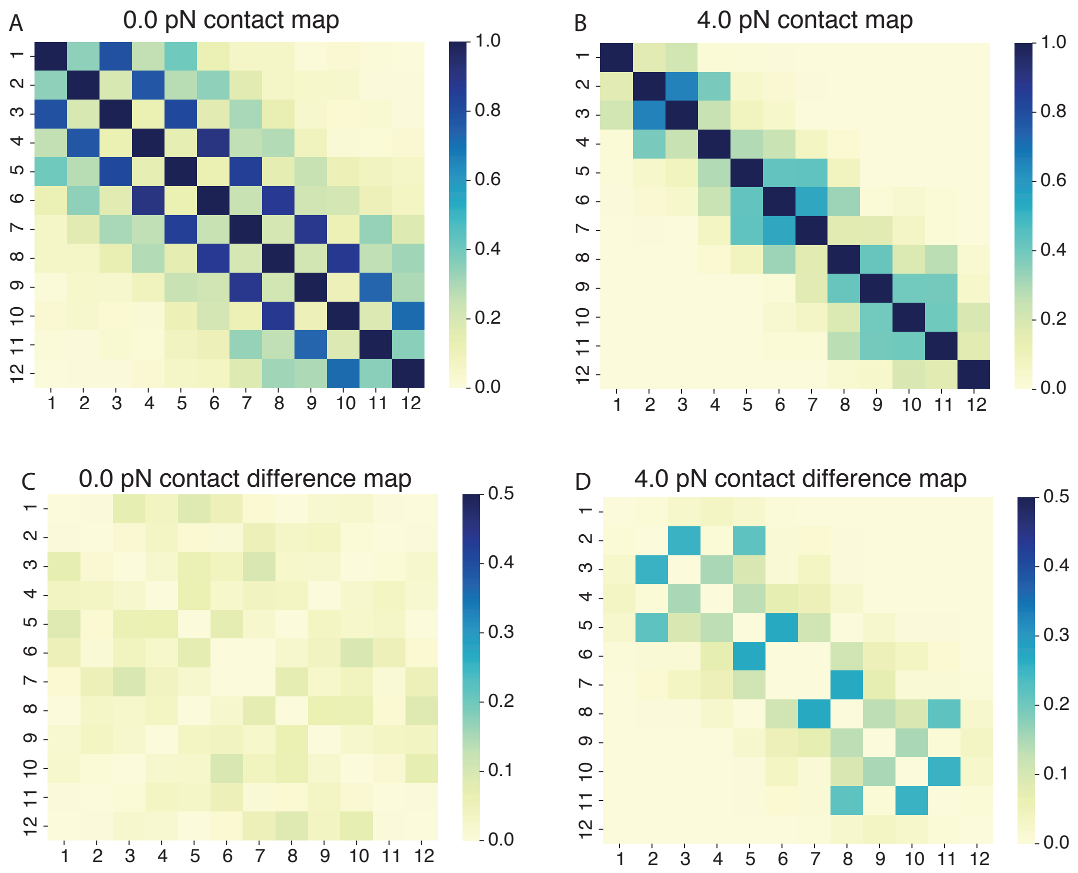

Figure S10: The ensemble of simulated chromatin configurations at different forces satisfy the C_2 symmetry. (A, B) Average nucleosome pair-wise contact maps computed using chromatin structures simulated with the presence of 0 and 4 pN force. The contact between nucleosome pairs (i, j) is defined as $c_{ij} = \left\langle \frac{1-\left(\frac{r_{ij}-d_0}{r_0}\right)^n}{1-\left(\frac{r_{ij}-d_0}{r_0}\right)^m} \right\rangle$ with $d_0 = 3$ nm, $r_0 = 8$ nm, $n = 6$, and $m = 12$. The angular brackets $\langle\cdot\rangle$ represent ensemble averaging. (C, D) Difference in contacts between pairs of nucleosomes defined as $\Delta c_{ij} = |c_{ij} - c_{13-i,13-j}|$. The difference in contacts was designed to examine the C_2 symmetry of the system. For example, we anticipate that for the 12mer, 1-2 nucleosomes should have comparable contacts as 11-12, 1-3 nucleosomes should have similar contacts as 10-12, etc. We note that the 12mer does not have translational symmetry, since n and $n+m$ nucleosomes are not identical due to the boundary effects and the finite length of chromatin.

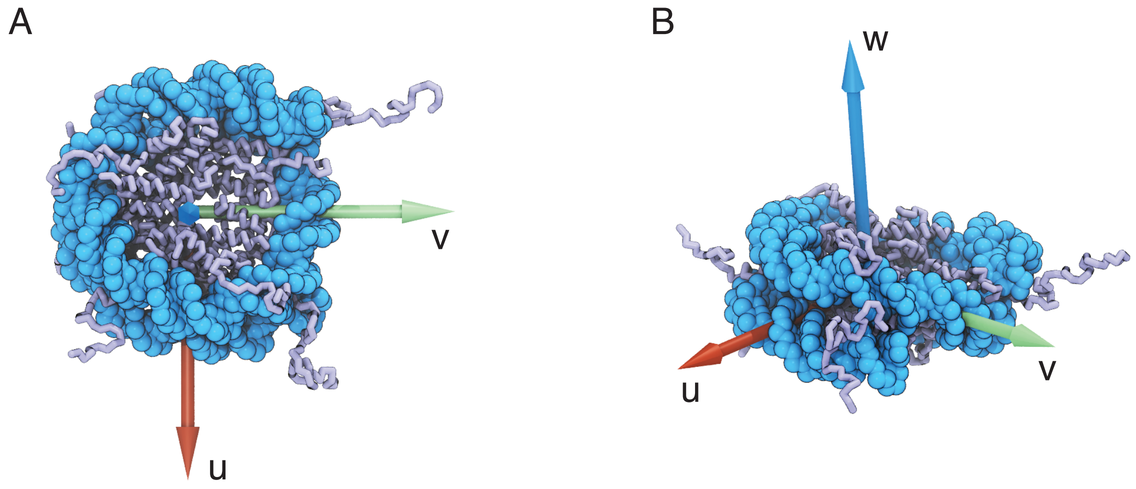

Figure S11: Illustration of the nucleosome coordinate system used to distinguish shearing and normal motions. The nucleosome is shown in the coarse-grained representation derived from the crystal structure (PDB ID: 1KX5).[S8] The origin of the coordinate system is defined as the center of residues 63-120, 165-217, 263-324, 398-462, 550-607, 652-704, 750-811, and 885-949. The red arrow points from the origin to the center of residues 63-120, 165-217, 750-811, and 885-949. The green arrow points towards the nucleosome dyad defined as the center of residues 81-131 and 568-618. The blue arrow is defined as the cross product of vectors along the red and the green arrows. See text *Section: Decomposing Inter-nucleosome Distances into Shear and Normal Motions* for further discussions.

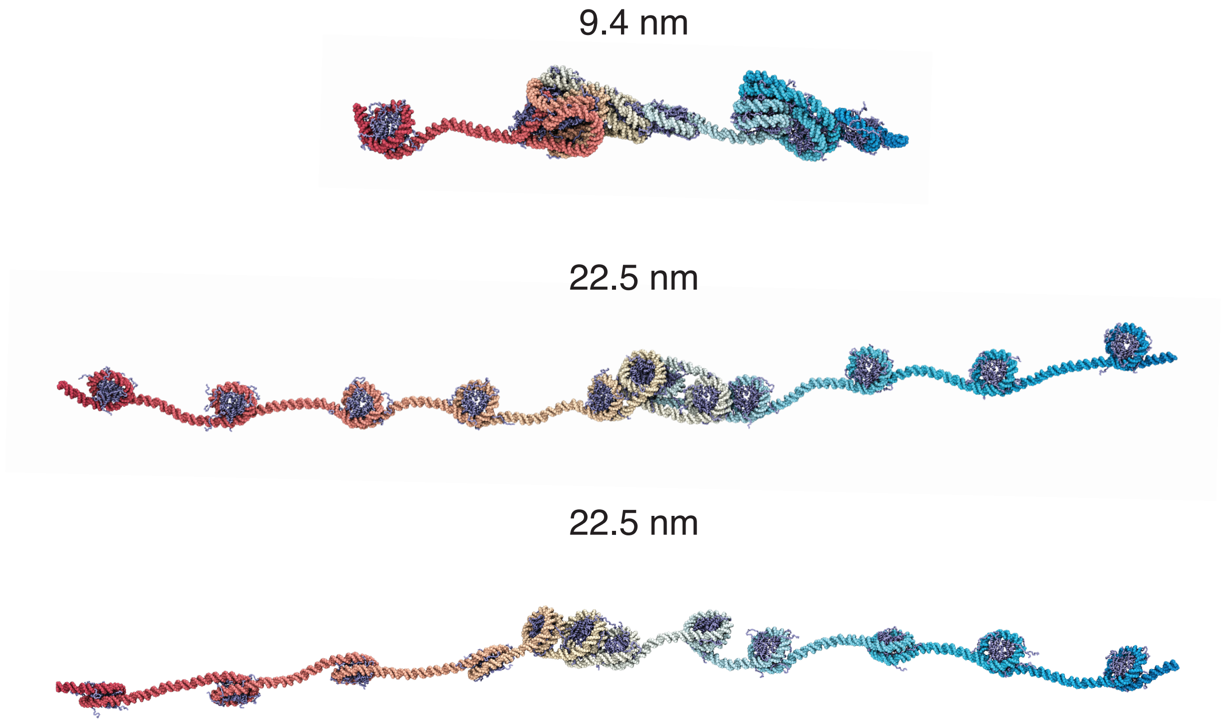

Figure S12: Additional representative chromatin structures at smaller and larger distances than the average extension at 4 pN force. The end-to-end distances are provided above the structures.

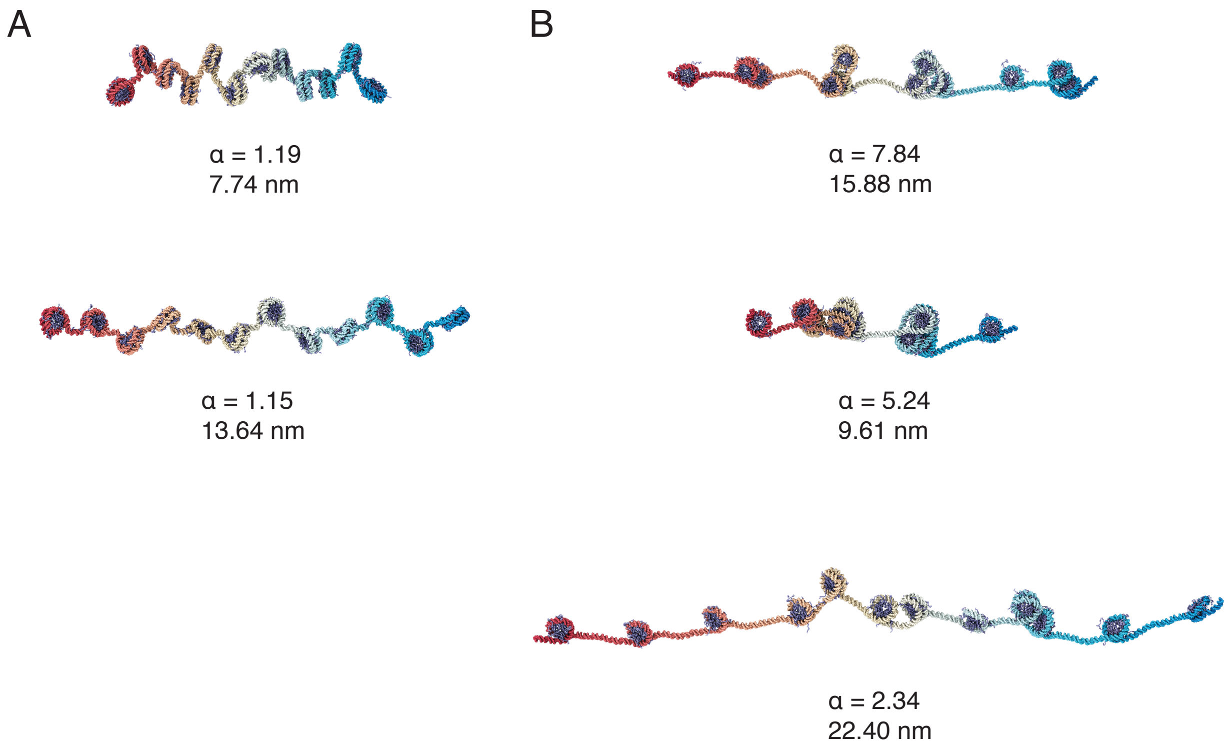

Figure S13: Simulations initialized from uniform chromatin configurations produce clutched structures. See text *Section: Simulations starting from uniformly extended chromatin configurations* for additional simulation details. (A) Illustration of the two uniformly extended configurations used to initialize the umbrella simulations. (B) Representative chromatin structures with different end-to-end distances per nucleosome produced by umbrella simulations. We selected configurations with the most likely α values. Numbers below the structures correspond to values for α and the end-to-end distance per nucleosome.

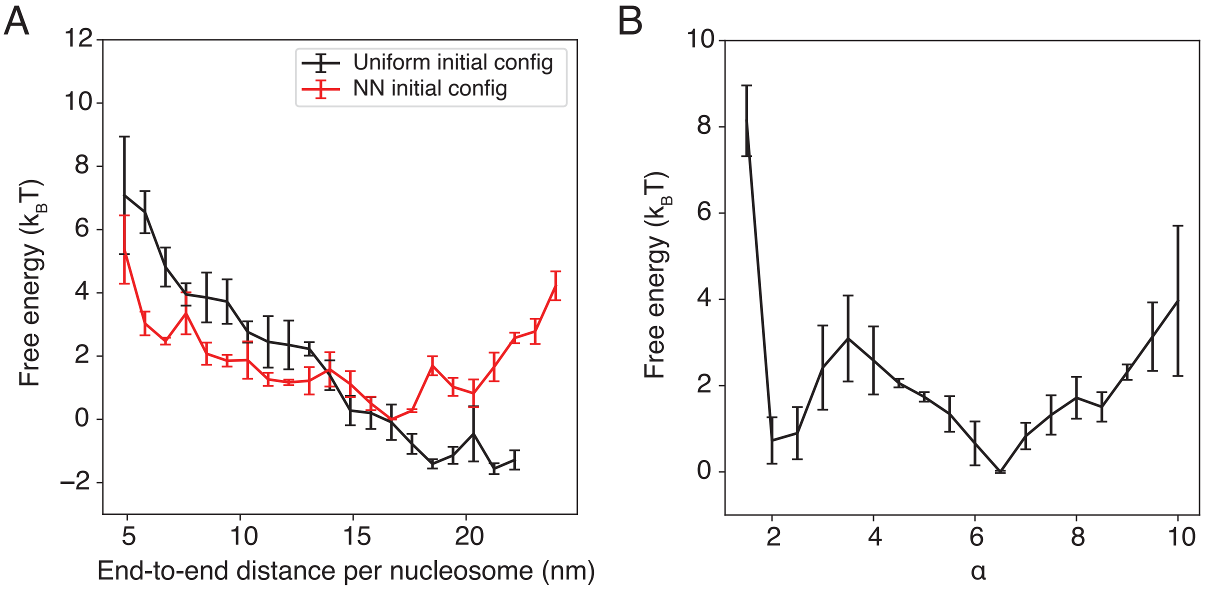

Figure S14: Simulations with uniform chromatin configurations reproduce findings presented in the main text. See text *Section: Simulations starting from uniformly extended chromatin configurations* for additional simulation details. (A) Comparison of the two free energy profiles as a function of end-to-end distance per nucleosome obtained from simulations with uniform chromatin configurations (black) and with configurations predicted by the neural network model (red). The red curve is identical to that presented in Figure 1C of the main text. The statistical equivalence of two independent sets of simulations initialized with different configurations within error bars supports the convergence of our results. We note that the residual differences between the two free energy profiles highlight the challenges of sampling chromatin configurations, which motivated our use of initial configurations predicted by the neural network model for simulations presented in the main text. (B) Free energy profile as a function of α. The global minimum at large α value supports the formation of clutched chromatin configurations.

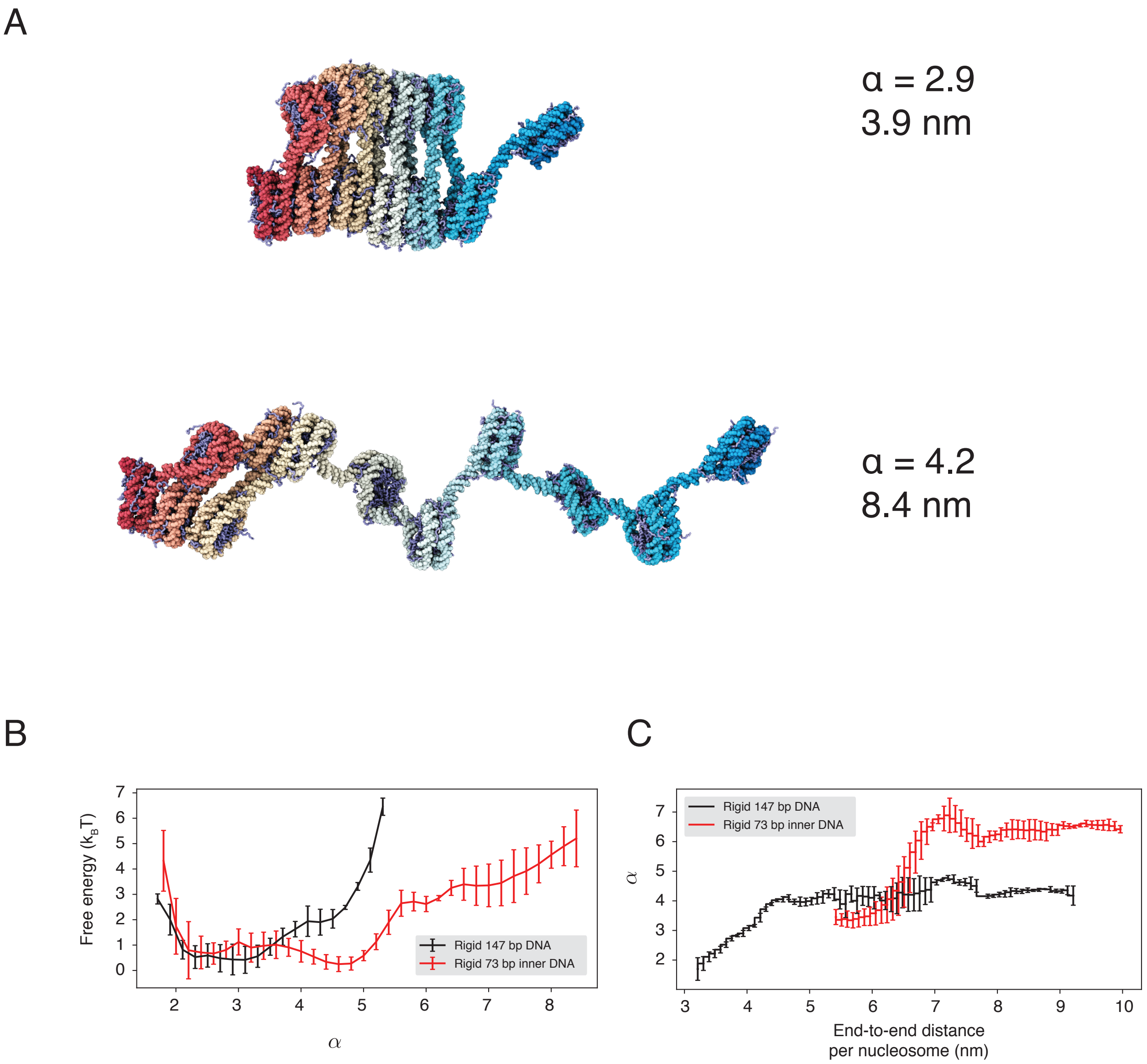

Figure S15: Restricting nucleosomal DNA unwrapping reduces clutch formation. See text *Section: Simulations with Fully Rigidified Nucleosomes* for additional simulation details. (A) Representative chromatin structures with different end-to-end distances produced by umbrella simulations. We selected configurations with the most likely α values. Numbers next to the structures correspond to values for α and the end-to-end distance per nucleosome. (B) Free energy profiles as a function of $\alpha = d^{\max}_{i,i+2}/d^{\min}_{i,i+2}$ calculated from simulations under 4 pN tension with the entire 147 bp nucleosomal DNA rigidified (black) and with only the inner 73 bp nucleosomal DNA rigidified (red). (C) The average value of α calculated as a function of the per-nucleosome DNA end-to-end distance from simulations under 4 pN tension with the entire 147 bp nucleosomal DNA rigidified (black) and with only the inner 73 bp nucleosomal DNA rigidified (red). Error bars are calculated from the standard deviation estimated via block averaging. For a better comparison between these two sets of simulations, we only show data with per-nucleosome end-to-end distance below 10 nm.

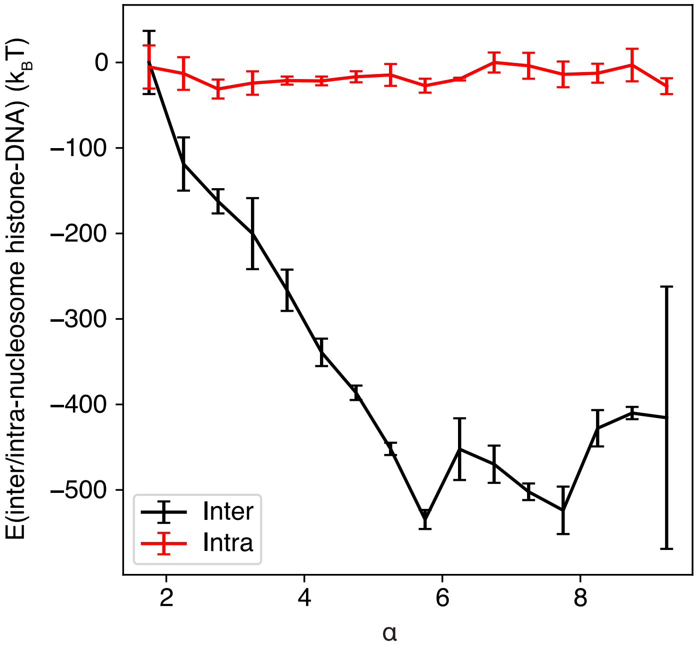

Figure S16: Correlation between $\alpha = d^{\max}_{i,i+2}/d^{\min}_{i,i+2}$, the ratio between maximum and minimum values of the 1-3 nucleosome stacking distance, and the inter or intra-nucleosome histone-DNA interaction energies. α was introduced to quantify the degree of irregularity in chromatin structure. As the name suggests, The intra-nucleosome energy (red) only accounts for the interactions between histone proteins and DNA segments from the same nucleosome, while the inter-nucleosome energy (black) quantifies interactions from different nucleosomes. The two curves were computed using data from simulations with the 4 pN force presented in the main text. They were shifted to set the maximum values as zero. The errorbars correspond to the standard deviation of the mean computed via block averaging.

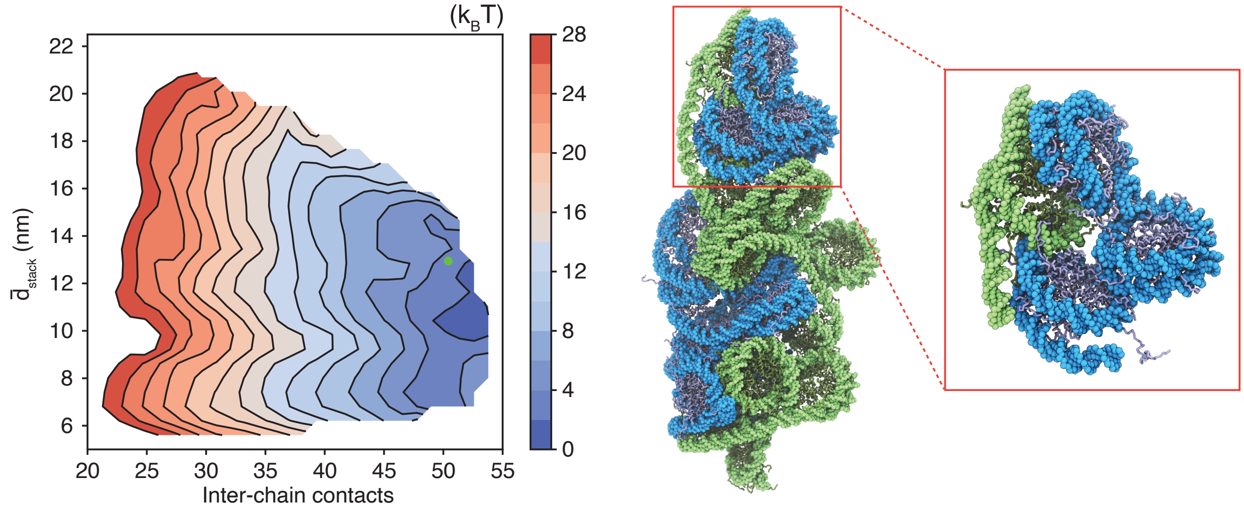

Figure S17: Representative structure of two contacting chromatin segments that adopt more extended configurations. Extension leads to more interdigitation between the two chains. The inset highlights the interactions between inter-chain nucleosomes. The free energy and collective variable value are indicated as the green dot in the free energy profile.

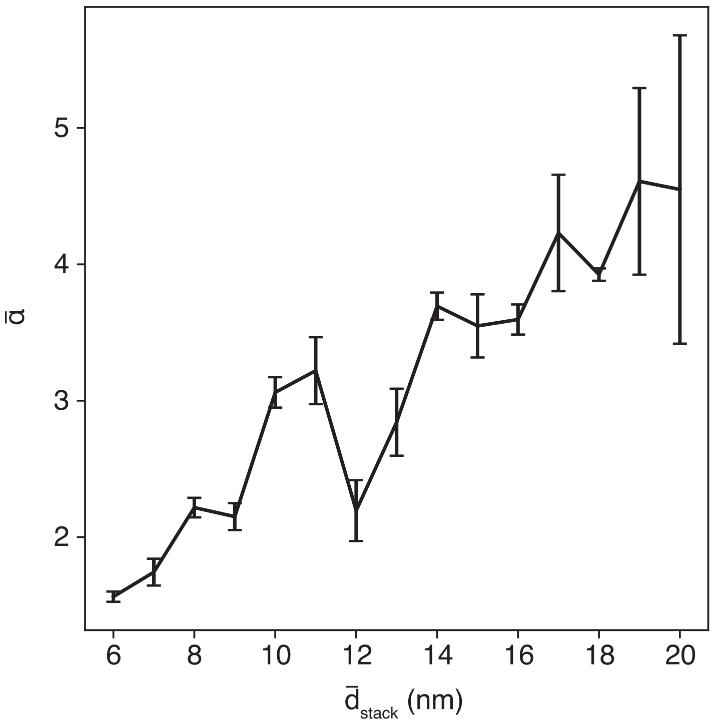

Figure S18: The average value of $\bar{\alpha}$ as function of $\bar{d}_{\text{stack}}$ determined using the same simulations presented in Figure 5 of the main text. $\alpha = d_{i,i+2}^{\text{max}}/d_{i,i+2}^{\text{min}}$ was introduced to quantify the degree of irregularity in chromatin structure. We averaged over two chromatin segments to define the mean value as $\bar{\alpha} = (\alpha_{\text{fiber 1}} + \alpha_{\text{fiber 2}})/2$. The errorbars measure the standard deviation of the mean and were estimated from three independent data blocks. This plot supports the formation of irregular chromatin configurations with nucleosome clutches (larger $\bar{\alpha}$ values) as chromatin extends to break stacking interactions (higher $\bar{d}_{\text{stack}}$ values).

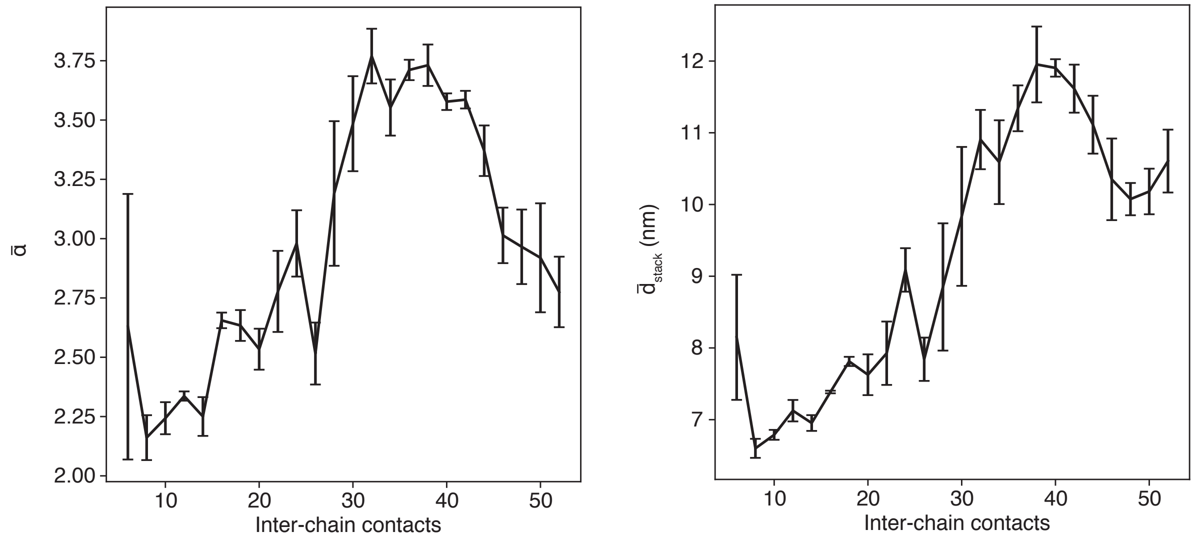

Figure S19: Average $\bar{\alpha}$ (Left) and $\bar{d}_{\text{stack}}$ (Right) as a function of inter-chain contact numbers determined using simulations presented in Figure 5 of the main text. The error bars measure the standard deviation of the mean and were estimated from three independent data blocks. The two plots support that chromatin become more irregular (larger $\bar{\alpha}$ values) and extended (larger $\bar{d}_{\text{stack}}$ values) as contacts form. The slight decrease in $\bar{\alpha}$ for very large contacts arises from chromatin compaction as seen in the drop for $\bar{d}_{\text{stack}}$. More contacts necessitate more compact chromatin configurations.

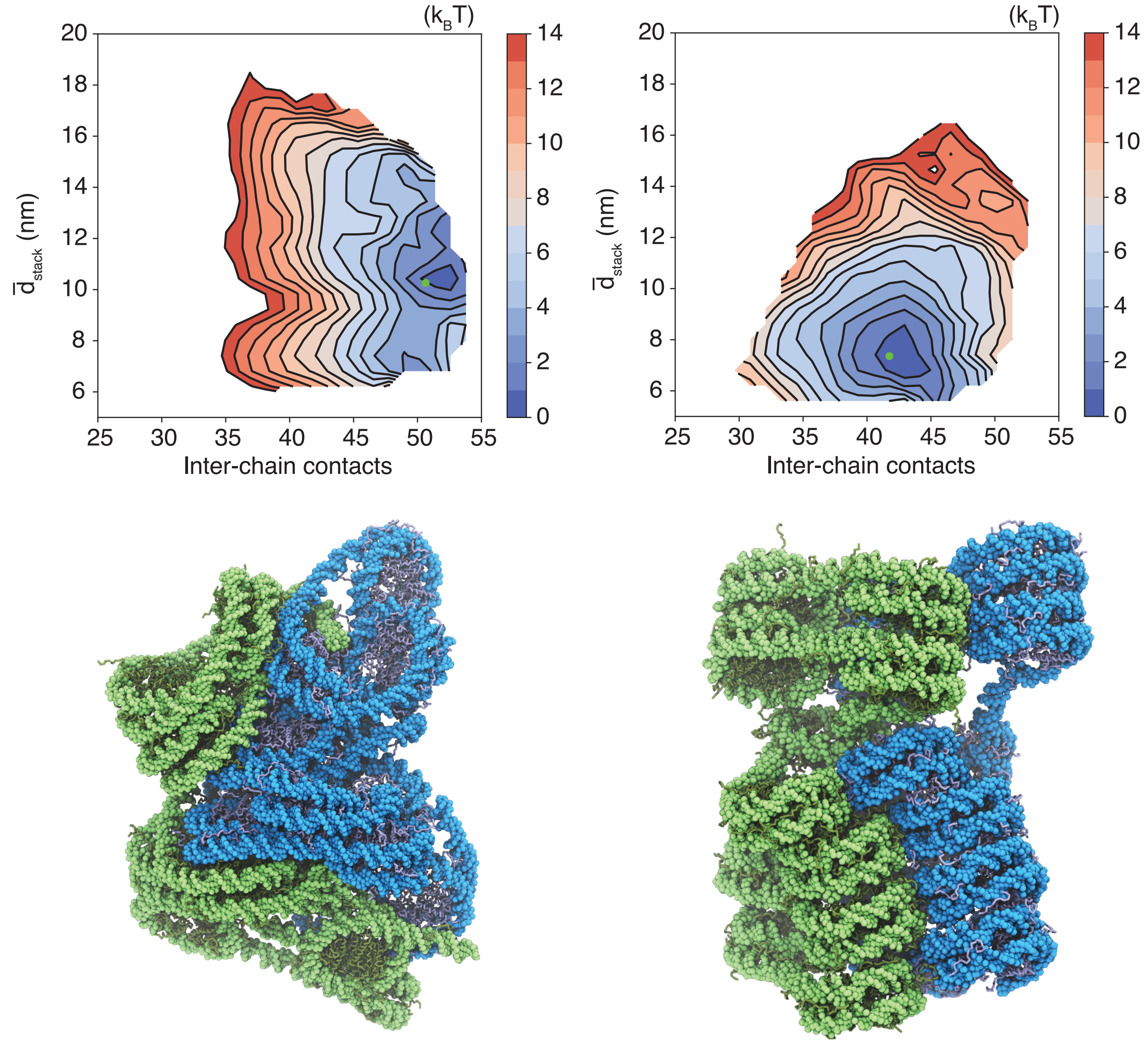

Figure S20: Free energy surface as a function of the inter-chain contacts and the average extension of the two 12mers determined with simulations that permit (left) or prohibit (right) outer nucleosomal DNA unwrapping. The left plot is identical to Figure 5A of the main text but with a different color scale. The right plot was computed with a new set of umbrella simulations in which the entire 147 bp nucleosomal DNA was rigified together with the histone core. Representative structures near the free energy minimum are shown below, with the collective variable values indicated as green dots in free energy surfaces. See *Section: Simulations with Fully Rigidified Nucleosomes* for simulation details.

(S1) Plimpton, S. Fast Parallel Algorithms for Short-Range Molecular Dynamics. *Journal of Computational Physics* **1995**, *117*, 1–19.

(S2) Bonomi, M.; Branduardi, D.; Bussi, G.; Camilloni, C.; Provasi, D.; Raiteri, P.; Donadio, D.; Marinelli, F.; Pietrucci, F.; Broglia, R. A.; Parrinello, M. PLUMED: A Portable Plugin for Free-Energy Calculations with Molecular Dynamics. *Computer Physics Communications* **2009**, *180*, 1961–1972.

(S3) Kumar, S.; Rosenberg, J. M.; Bouzida, D.; Swendsen, R. H.; Kollman, P. A. The Weighted Histogram Analysis Method for Free-Energy Calculations on Biomolecules. I. The Method. *J. Comput. Chem.* **1992**, *13*, 1011–1021.

(S4) Noel, J. K.; Levi, M.; Raghunathan, M.; Lammert, H.; Hayes, R. L.; Onuchic, J. N.; Whitford, P. C. SMOG 2: A Versatile Software Package for Generating Structure-Based Models. *PLoS Comput Biol* **2016**, *12*, e1004794.

(S5) Ding, X.; Vilseck, J. Z.; Brooks III, C. L. Fast Solver for Large Scale Multistate Bennett Acceptance Ratio Equations. *J. Chem. Theory Comput.* **2019**, *15*, 799–802.

(S6) Song, F.; Chen, P.; Sun, D.; Wang, M.; Dong, L.; Liang, D.; Xu, R.-M.; Zhu, P.; Li, G. Cryo-EM Study of the Chromatin Fiber Reveals a Double Helix Twisted by Tetranucleosomal Units. *Science* **2014**, *344*, 376–380.

(S7) Schalch, T.; Duda, S.; Sargent, D. F.; Richmond, T. J. X-Ray Structure of a Tetranucleosome and Its Implications for the Chromatin Fibre. *Nature* **2005**, *436*, 138–141.

(S8) Davey, C. A.; Sargent, D. F.; Luger, K.; Maeder, A. W.; Richmond, T. J. Solvent Mediated Interactions in the Structure of the Nucleosome Core Particle at 1.9 Å Resolution. *J. Mol. Biol.* **2002**, *319*, 1097–1113.

(S9) Lu, X.-J. 3DNA: A Software Package for the Analysis, Rebuilding and Visualization of

Three-Dimensional Nucleic Acid Structures. *Nucleic Acids Research* **2003**, *31*, 5108–5121.

(S10) Michaud-Agrawal, N.; Denning, E. J.; Woolf, T. B.; Beckstein, O. MDAnalysis: A Toolkit for the Analysis of Molecular Dynamics Simulations. *J Comput Chem* **2011**, *32*, 2319–2327.

(S11) Gowers, R.; Linke, M.; Barnoud, J.; Reddy, T.; Melo, M.; Seyler, S.; Domański, J.; Dotson, D.; Buchoux, S.; Kenney, I.; Beckstein, O. MDAnalysis: A Python Package for the Rapid Analysis of Molecular Dynamics Simulations. Python in Science Conference. Austin, Texas, 2016; pp 98–105.

(S12) Theobald, D. L. Rapid Calculation of RMSDs Using a Quaternion-Based Characteristic Polynomial. *Acta Crystallogr A* **2005**, *61*, 478–480.

(S13) Liu, P.; Agrafiotis, D. K.; Theobald, D. L. Fast Determination of the Optimal Rotational Matrix for Macromolecular Superpositions. *J. Comput. Chem.* **2010**, *31*, 1561–1563.

(S14) Koslover, E. F.; Fuller, C. J.; Straight, A. F.; Spakowitz, A. J. Local Geometry and Elasticity in Compact Chromatin Structure. *Biophys J* **2010**, *99*, 3941–3950.

(S15) Wales, D. J. *Energy Landscapes*; Cambridge Molecular Science; Cambridge University Press: Cambridge, UK ; New York, 2003.

(S16) Ding, X.; Lin, X.; Zhang, B. Stability and Folding Pathways of Tetra-Nucleosome from Six-Dimensional Free Energy Surface. *Nat. Commun.* **2021**, *12*, 1–9.

(S17) Freeman, G. S.; Hinckley, D. M.; Lequieu, J. P.; Whitmer, J. K.; de Pablo, J. J. Coarse-Grained Modeling of DNA Curvature. *The Journal of Chemical Physics* **2014**, *141*, 165103.

(S18) Clementi, C.; Nymeyer, H.; Onuchic, J. N. Topological and Energetic Factors: What Determines the Structural Details of the Transition State Ensemble and "En-Route" Intermediates for Protein Folding? An Investigation for Small Globular Proteins. *J Mol Biol* **2000**, *298*, 937–953.

(S19) Noel, J. K.; Whitford, P. C.; Onuchic, J. N. The Shadow Map: A General Contact Definition for Capturing the Dynamics of Biomolecular Folding and Function. *J. Phys. Chem. B* **2012**, *116*, 8692–8702.

(S20) Miyazawa, S.; Jernigan, R. L. Estimation of Effective Interresidue Contact Energies from Protein Crystal Structures: Quasi-Chemical Approximation. *Macromolecules* **1985**, *18*, 534–552.

(S21) Zhang, B.; Zheng, W.; Papoian, G. A.; Wolynes, P. G. Exploring the Free Energy Landscape of Nucleosomes. *J. Am. Chem. Soc.* **2016**, *138*, 8126–8133.

(S22) Lequieu, J.; Córdoba, A.; Schwartz, D. C.; de Pablo, J. J. Tension-Dependent Free Energies of Nucleosome Unwrapping. *ACS Cent. Sci.* **2016**, *2*, 660–666.

(S23) Parsons, T.; Zhang, B. Critical Role of Histone Tail Entropy in Nucleosome Unwinding. *J. Chem. Phys.* **2019**, *150*, 185103.

(S24) Freeman, G. S.; Lequieu, J. P.; Hinckley, D. M.; Whitmer, J. K.; De Pablo, J. J. DNA shape dominates sequence affinity in nucleosome formation. *Physical Review Letters* **2014**, *113*, 168101.

(S25) Moller, J.; Lequieu, J.; de Pablo, J. J. The Free Energy Landscape of Internucleosome Interactions and Its Relation to Chromatin Fiber Structure. *ACS Cent. Sci.* **2019**, *5*, 341–348.

(S26) Funke, J. J.; Ketterer, P.; Lieleg, C.; Schunter, S.; Korber, P.; Dietz, H. Uncovering the forces between nucleosomes using DNA origami. *Science advances* **2016**, *2*, e1600974.

(S27) Buning, R.; Kropff, W.; Martens, K.; van Noort, J. spFRET reveals changes in nucleosome breathing by neighboring nucleosomes. *Journal of Physics: Condensed Matter* **2015**, *27*, 064103.

(S28) Machado, M. R.; Barrera, E. E.; Klein, F.; Sóñora, M.; Silva, S.; Pantano, S. The SIRAH 2.0 force field: altius, fortius, citius. *Journal of Chemical Theory and Computation* **2019**, *15*, 2719–2733.

(S29) Woods, D. C.; Rodríguez-Ropero, F.; Wereszczynski, J. The Dynamic Influence of Linker Histone Saturation within the Poly-Nucleosome Array. *Journal of Molecular Biology* **2021**, *433*, 166902.

(S30) Meng, H.; Andresen, K.; Van Noort, J. Quantitative Analysis of Single-Molecule Force Spectroscopy on Folded Chromatin Fibers. *Nucleic Acids Res.* **2015**, *43*, 3578–3590.

(S31) Kingma, D. P.; Ba, J. Adam: A method for stochastic optimization. *arXiv preprint arXiv:1412.6980* **2014**,

(S32) Jumper, J.; Evans, R.; Pritzel, A.; Green, T.; Figurnov, M.; Ronneberger, O.; Tunyasuvunakool, K.; Bates, R.; Žídek, A.; Potapenko, A., et al. Highly accurate protein structure prediction with AlphaFold. *Nature* **2021**, *596*, 583–589.

(S33) Kaczmarczyk, A.; Meng, H.; Ordu, O.; van Noort, J.; Dekker, N. H. Chromatin Fibers Stabilize Nucleosomes under Torsional Stress. *Nat. Commun.* **2020**, *11*, 1–12.

(S34) Correll, S. J.; Schubert, M. H.; Grigoryev, S. A. Short nucleosome repeats impose rotational modulations on chromatin fibre folding. *The EMBO journal* **2012**, *31*, 2416–2426.

(S35) Mihardja, S.; Spakowitz, A. J.; Zhang, Y.; Bustamante, C. Effect of force on mononucleosomal dynamics. *Proceedings of the National Academy of Sciences* **2006**, *103*, 15871–15876.

(S36) Wei, S.; Falk, S. J.; Black, B. E.; Lee, T.-H. A novel hybrid single molecule approach reveals spontaneous DNA motion in the nucleosome. *Nucleic acids research* **2015**, *43*, e111–e111.

(S37) Chien, F.-T.; Van Der Heijden, T. Characterization of nucleosome unwrapping within chromatin fibers using magnetic tweezers. *Biophysical journal* **2014**, *107*, 373–383.

(S38) Gemmen, G. J.; Sim, R.; Haushalter, K. A.; Ke, P. C.; Kadonaga, J. T.; Smith, D. E. Forced unraveling of nucleosomes assembled on heterogeneous DNA using core histones, NAP-1, and ACF. *Journal of molecular biology* **2005**, *351*, 89–99.
